# Exploring Single-Probe Single-Cell Mass Spectrometry: Current Trends and Future Directions

**DOI:** 10.1021/acs.analchem.4c06824

**Published:** 2025-02-25

**Authors:** Deepti Bhusal, Shakya Wije Munige, Zongkai Peng, Zhibo Yang

**Affiliations:** † Department of Chemistry and Biochemistry, University of Oklahoma, Norman, Oklahoma 73019, United States; ‡ Stephenson Cancer Center, University of Oklahoma Health Sciences Center, Oklahoma City, Oklahoma 73104, United States

## Abstract

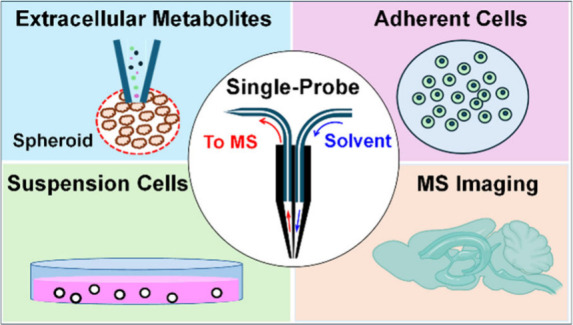

The Single-probe single-cell mass spectrometry (SCMS) is an innovative analytical technique designed for metabolomic profiling, offering a miniaturized, multifunctional device capable of direct coupling to mass spectrometers. It is an ambient technique leveraging microscale sampling and nanoelectrospray ionization (nanoESI), enabling the analysis of cells in their native environments without the need for extensive sample preparation. Due to its miniaturized design and versatility, this device allows for applications in diverse research areas, including single-cell metabolomics, quantification of target molecules in single cell, MS imaging (MSI) of tissue sections, and investigation of extracellular molecules in live single spheroids. This review explores recent advancements in Single-probe-based techniques and their applications, emphasizing their potential utility in advancing MS methodologies in microscale bioanalysis.

## Introduction

In recent years, single-cell analysis has emerged as a transformative approach in analytical chemistry, offering unprecedented insights into cellular heterogeneity and enabling the study of intricate biological processes at the cellular level. Cellular heterogeneity is a common feature in almost all biological systems. Beyond genetic differences, it can also arise from nongenetic mechanisms, where cells with similar genotypes exhibit distinct morphological and phenotypical traits.^1,2^ This heterogeneity may stem from diverse factors such as the cell cycle, stochastic variations in gene expression, and interactions with the surrounding microenvironment.^3−5^ In addition to studies of cell heterogeneity, single-cell analysis is needed for other applications, including research of rare cells (e.g., cancer stem cells),^6−8^ development biology (e.g., cell changes during embryonic development),^9−11^ and personalized medicine (e.g., analyzing individual cells from a patient for personalized treatment).^12−16^

Owing to its multiple advantages (e.g., high sensitivity, high accuracy, and broad molecular coverage), mass spectrometry (MS) is regarded as one of the most important techniques for molecular analysis. With the recent advancement, a variety of different single-cell MS (SCMS) methods have been established as powerful tools to analyze large (e.g., proteins) and small (e.g., metabolites) within individual cells.^17,18^ Metabolomics is the study of metabolites, which are broadly defined small molecules with molecular weight <1500 amu, such as lipids, fatty acids, peptides, amino acids, nucleic acids, sugars, and organic acids.^19,20^ The Single-probe, which is a microscale sampling and ionization device, can be coupled to a mass spectrometer for SCMS metabolomics studies of live single cells without complex sample preparation or labeling. The interest in the Single-probe SCMS stems from its potential to deepen our understanding of single-cell metabolism, driving both fundamental research and clinical applications forward. Notable innovations include integration with microscopy methods (e.g., fluorescence microscopy) and combination with chemical reactions, greatly extending the application of this technique. As a multifunctional device, the Single-probe can be coupled to mass spectrometry for studies in multiple different areas, including single-cell metabolomics,^21,22^ MS imaging (MSI) of tissue slices,^23−27^ and the analysis of extracellular molecules in live single spheroids,^28^ within ambient conditions. Additionally, we have developed other techniques, such as the T-probe^29,30^ and micropipette capillary,^31^ to facilitate SCMS measurements. As a robust technique, the Single-probe SCMS method has been used in a variety of fundamental studies such as analysis of cellular heterogeneity,^32−34^ investigation of cell–cell interactions,^35^ detection of signaling molecules,^8,36^ and environmental influences on cell metabolism.^37,38^ In addition, the Single-probe SCMS technique has promising clinical applications, as it has been implemented to detect and quantify drug molecules in single cells,^34,39−42^ characterize cancer stem cells,^8^ investigate drug resistance and metabolic responses to drugs,^43−46^ and study human diseases.^38,47^ In addition to mammalian cells, the Single-probe SCMS technique has been used to study plant cells.^48,49^ This Review aims to explore recent advancements and applications of the Single-probe SCMS techniques, underscoring its growing significance in analytical chemistry. As this technology continues to evolve, it promises to usher in a new era in microscale bioanalysis, enabling unprecedented insights into complex biological systems at the cellular and tissue levels.

## OVERVIEW OF CURRENT SCMS TECHNIQUES

The current SCMS techniques can broadly be classified into two categories based on their sampling and ionization environments: vacuum-based and ambient methods.^18,50,51^

### Vacuum-Based Ionization Techniques

These methods are known for their high sensitivity and spatial resolution, and they are well-suited for single-cell analysis.^52^ However, these experiments require complex sample preparation, such as dehydration and matrix application, to facilitate ion generation through lasers or ion beams.^52^ Key single-cell MS technologies in this category include secondary ion mass spectrometry (SIMS), gas cluster ion beam (GCIB), matrix-assisted laser desorption/ionization (MALDI), and matrix-free laser desorption/ionization (LDI).

#### SIMS-Based Methods

SIMS, originally demonstrated by Herzog and Biehböck in 1949,^53^ evolved for single-cell analysis in the 1960s.^54,55^ SIMS provides sensitive analysis of surface compositions by sputtering analytes with a focused primary ion beam (e.g., ^16^O^–^, ^16^O_2_^+^, and ^40^Ar^+^), which generates secondary ions from surface molecules. The established SIMS methods for single-cell analysis include TOF-SIMS,^56−58^ nanoSIMS,^59,60^ and the newer GCIB-SIMS.^61,62^ These techniques render high spatial resolution (e.g., 50 nm spatial resolution can be achieved using nanoSIMS); however, challenges remain for analyzing small biological samples such as single cells.^63^ These challenges include the high vacuum requirement, low ionization efficiency for biomolecules, and complex data analysis due to extensive fragmentation from high-energy ion bombardment.^61^ Advances in the ion beam source, such as the GCIB, have been introduced to mitigate fragmentation.^61,62^

#### Laser Desorption/Ionization (LDI)-Based Methods

These techniques employ laser beams at specific wavelengths to irradiate the sample surface, desorbing and ionizing molecules. Although laser technology emerged in 1960 with Maiman’s invention, the potential of LDI for MS was only realized in the 1980s, as early LDI methods could ionize only molecules absorbing specific laser wavelengths.^64^ Key LDI approaches include matrix-assisted laser desorption/ionization (MALDI-MS) and matrix-free LDI. MALDI, a soft ionization method, significantly enhances the ionization efficiency of large biomolecules such as proteins and polymers.^65,66^ In a MALDI experiment, an organic matrix compound with strong UV absorption assists in laser absorption, enabling efficient energy transfer to the analytes. Ionization occurs through the interactions between the analyte and ionized matrix molecules, but the high vacuum environment required to prevent atmospheric interference may lead to molecular delocalization and other sample alterations. Additionally, matrix compounds often interfere with detection of low-molecular-weight compounds (<1000 *m*/*z*), complicating studies on small molecules like metabolites and drug compounds.^67−69^ To minimize interference with matrix molecules, alternative MS techniques, such as matrix-free laser desorption/ionization MS (LDI-MS)^70,71^ and label-assisted laser desorption/ionization MS (LALDI-MS),^72,73^ have been developed. These methods are particularly useful for analyzing relatively large cells, including plant^73^ and algae cells.^74^ In LALDI-MS, specific functional groups (e.g., fluorophores or polyaromatic structures) are used to label target molecules (e.g., peptides) to enable their desorption and ionization when exposed to soft lasers operating at visible wavelengths. While some LDI-based methods are described as matrix-free, it is often challenging to completely avoid the use of assistive molecules (e.g., 1,5-diaminonaphthalene) when studying biological samples. This is largely due to the complexity of biomolecules, which often demand varied levels of desorption and ionization energy.^69^ While these vacuum-based SCMS methods minimize interference from experimental contaminants, allowing for enhanced detection sensitivity and high throughput analysis, they require nontrivial sample preparation as well as preclude the analysis of live cells due to the extensive pretreatments involved.^52,75^

### Ambient-Based Sampling and Ionization Techniques

Compared with vacuum-based techniques, ambient SCMS methods offer greater flexibility, enabling in-situ analysis of cells within their native or near-native environments. This capability makes ambient SCMS more suitable for live cell studies. However, the sensitivity of ambient techniques is typically lower than that of vacuum-based methods, due to ionization efficiency caused by interference from matrix molecules.^76^ Additionally, the throughput of most ambient-based methods tends to be lower, limiting their applicability for studies requiring a large number of cells. Despite these challenges, significant advancements have been made to improve the throughput of ambient SCMS techniques, making them a valuable tool for minimally invasive live-cell analysis.^76^ Many ambient SCMS techniques typically employ physical probes, lasers, or charged solvent droplets to facilitate analyte sampling and ionization.

According to the methods used for sampling contents from single cells, we classified ambient SCMS techniques into three categories: direct suction by microprobes, microextraction by probe with solid or liquid phase, and direct desorption and ionization.^43,76^ The first two categories primarily use probe-based approaches. Due to the small size of single cells, often only a few micrometers, traditional sampling and preparation techniques from bulk analyses are not applicable. Microprobes were introduced to meet the specific requirements of single-cell analysis.^76^

#### Direct Suction by Microprobes

The concept of the microprobe was initially proposed by Masujima in 1999,^77^ leading to the first ambient SCMS experiment in 2008 using live single-cell video mass spectrometry (Video-MS).^78^ In these early experiments, cells were monitored using a video microscope, and a gold-coated capillary nanoelectrospray ionization (nanoESI) emitter (tip size is ∼1–2 μm) was employed as a micropipette to extract cell contents.^78^ The same nanoESI emitter was then used for ionization in MS analysis. This technique has been applied to study plant cells, quantify analytes in live SCMS, and integrate with fluorescence imaging, laser microscopy, and microdroplet array systems.^78−81^ Vertes et al. integrated the capillary microsampling system^82^ with ion mobility MS to identify metabolites in single human hepatocytes.^83^ Additionally, this system has been applied to analyze neurons of the mollusk *Lymnaea stagnalis*.^84^ When combined with fluorescence microscopy, specific subcellular components (e.g., cytoplasm and nucleus) can be selected for analysis. The pressure probes^85^ facilitated direct sample injection using an internal electrode capillary,^86^ reducing the need for extensive sample preparation. Pico-ESI capabilities enabled these probes to be operated efficiently under ambient conditions. Other direct-suction methods, including nanopipettes,^87^ micropipettes,^31^ and T-probes,^29,17^ have also been developed for ambient single-cell analysis.

#### Microextraction by Probe with Solid or Liquid Phase

These methods for single-cell analysis are divided into solid–liquid and liquid–liquid microextractions.^76^ In solid–liquid microextraction,^88^ a surface-treated metal needle is introduced into a single cell to extract and enrich analytes, which are subsequently analyzed by a mass spectrometer under ambient conditions. Techniques, such as probe electrospray ionization,^89^ direct sampling probes,^90,91^ and surface-coated probe nanoESI-MS,^92−95^ are examples of solid–liquid microextraction developed for single-cell analysis. On the other hand, liquid–liquid microextraction employs organic solvents (e.g., methanol and acetonitrile) for extraction. These methods generally do not require additional solvents for MS analysis, leading to a higher throughput compared to solid–liquid techniques. In liquid–liquid extraction, a capillary is typically used to introduce the solvent, which then carries dissolved analytes to the mass spectrometer. Major liquid–liquid microextraction methods include nanomanipulation, nano-DESI, and the Single-probe MS. In nanomanipulation coupled nanospray MS, introduced by Phelps et al.,^92^ a quartz probe punctures the cell membrane, and a nanoESI emitter is used to extract analytes. Nano-DESI, developed by the Laskin group in 2012,^96^ utilizes a primary capillary to deliver solvent to the sample and a secondary capillary for solution extraction and ionization. Originally designed for MS imaging, nano-DESI was later adapted by Lanekoff et al. for single-cell analysis.^97^ In the subsequent sections of this Review, we will provide a detailed discussion on the applications of the Single-probe techniques in single-cell analysis.

#### Direct Desorption and Ionization

These techniques involve the application of laser energy, charged particles, or high electric fields to facilitate analyte desorption/ionization from individual cells, producing gas-phase ions suitable for MS detection under ambient and open-air conditions. Approaches include desorption electrospray ionization (DESI)-MS,^98,99^ easy ambient sonic-spray ionization, drop-on-demand inkjet printing with probe electrospray ionization (PESI)-MS, and laser-based methods such as laser ablation electrospray ionization (LAESI),^100,101^ laser desorption/ionization droplet delivery (LDIDD),^102^ and atmospheric-pressure MALDI (AP-MALDI).^103^

## SINGLE-PROBE SCMS TECHNIQUES

The Single-probe is a sophisticated analytical tool composed of several integral components that work together to facilitate microscale bioanalysis. Here, we provide a review of its fabrication as well as applications in SCMS analysis of small molecules (i.e., semiquantitative analysis, quantitative analysis, integration with chemical reactions, evaluation of single cell sample preparation, and combined advanced data analysis), MSI of biological tissues, MS analysis of extracellular molecules in live spheroids, and other studies performed using the Single-probe-based techniques.

### Single-Probe Fabrication

**Figure 1:**
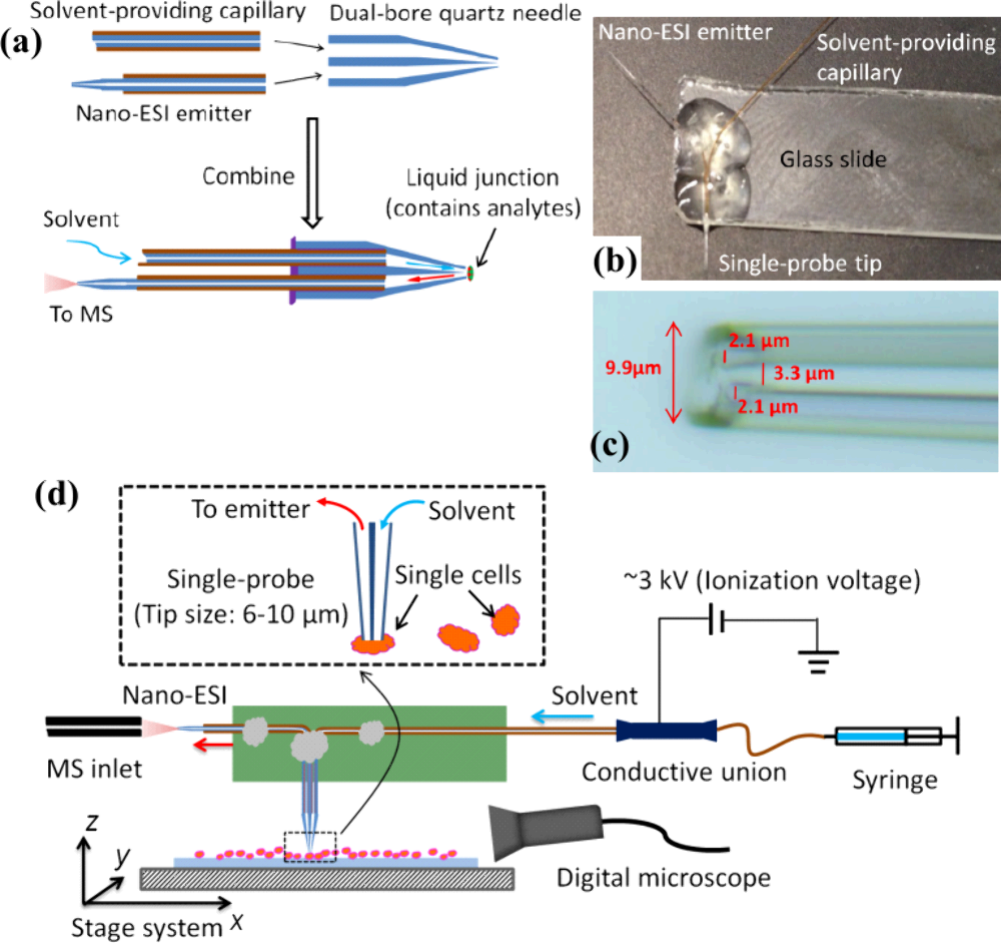
Single-probe SCMS. (a) Fabrication steps of the Single-probe; (b) photograph of a Single-probe; (c) zoomed-in view (40× magnification) of the Single-probe tip with measurements from a calibrated digital microscope; (d) schematic of the Single-probe setup for SCMS analysis. [Reproduced with permission from ref (21). Copyright 2014, American Chemical Society, Washington, DC.]

The fabrication of the Single-probe (Figures 1a–c) has been thoroughly described in our previous studies.^21,25,34,39,104^ This assembly includes three key elements: a laser-pulled dual-bore quartz needle, a solvent providing silica capillary, and a nanoelectrospray ionization (nano-ESI) emitter that efficiently ionizes the extracted metabolite. The fabrication of the single probe begins with the precise shaping of a dual-bore quartz needle (outer diameter (OD) 500 μm; inner diameter (ID) 127 μm, Friedrich & Dimmock, Inc., Millville, NJ, USA)) using a laser pipet puller (Model P-2000, Sutter Instrument CO., Novato, CA). This pulling process creates a fine, tapered structure in the quartz needle. Following this step, a fused silica capillary (outer diameter 105 μm, inner diameter 40 μm, Polymicro Technologies, Phoenix, AZ) is embedded into one bore of the pulled quartz needle to serve as the solvent delivery channel. Additionally, a nano-ESI emitter is positioned within the other bore. The nano-ESI emitter is formed by heating a similar fused silica capillary with a butane micro torch to achieve a sharp, functional tip for effective ionization. To secure both the capillary and the nano-ESI emitter within the dual-bore needle, UV-curing epoxy (Prime Dental, Item No. 006.030, Chicago, IL) is applied to glue these parts and is then cured under a UV LED lamp.

To ensure the ease of use and stability of the device during sampling, the Single-probe is mounted on a microscope glass slide using standard epoxy adhesive (Part No. 20945, ITW Devcon, Inc., Danvers, MA) (Figure 1b). A Conductive MicroTight Union (M-539, IDEX Health & Science, LLC) connects the fused silica capillary (ID: 50 μm, OD: 150 μm) to the solvent-providing capillary. A PEEK tubing (F-181 and F-380, IDEX Health & Science, LLC) is used as the sleeve of the fused silica capillary to ensure a tight connection. The ionization voltage is applied to the union instead of the nano-ESI emitter, enabling efficient solvent delivery and ionization. To construct a functioning setup, the Single-probe is combined with other components, including a motorized XYZ-stage (CONEX- MFACC, Newport Corp., Irvine, CA), a manual XYZ-translation stage (Compact Dovetail XYZ Linear Stage, Newport Corp., Irvine, CA), a stereomicroscope (Supereyes T004 Digital Microscope, Shenzhen D&F Co., Ltd., Shenzhen, China), and a flexible connector (MXB-3 h, Siskiyou Corp., Grants Pass, OR). All components are integrated on an optical board (Thorlabs Inc., Newton, NJ, US) interfaced with the mass spectrometer (Thermo LTQ Orbitrap XL mass spectrometer, Thermo Fisher Scientific, Inc., Waltham, MA) (Figure 1d).

### Single-Probe SCMS Studies

#### Single-Probe SCMS in Semiquantitative Studies

The Single-probe SCMS technique has been used to characterize cellular metabolites through semiquantitative analysis, in which ion intensities of metabolites are normalized to the total ion current (TIC) as commonly performed in MS studies. The Single-probe semiquantitative approaches have been used for uncovering molecular diversity and cellular heterogeneity.^8,34,35,38,41−43,46,47,49,105^ This section outlines the progression of semiquantitative applications of the Single-probe SCMS technique in single-cell metabolomics, highlighting its evolution across diverse biological contexts.

One of the earliest qualitative applications was demonstrated in 2018 by Sun et al., who employed the Single-probe SCMS to investigate intracellular metabolite changes in *Scrippsiella trochoidea*, a marine dinoflagellate, under various environmental conditions.^49^ Bulk filtration techniques are predominantly used to assess the physiological responses of microbial populations to environmental changes. The Single-probe SCMS technique provided profiles of intracellular metabolites in these single marine algae cells altered by different conditions such as light variation and nitrogen limitation. This work is a showcase of the potential applications of single-cell metabolomics studies of marine algae cells’ responses to environmental stressors without extensive sample manipulation.

To further extend the scope of SCMS, a novel platform integrating a commercially available cell manipulation system with the Single-probe technique was developed, allowing for the analysis of suspended cells such as leukemia cells.^34,105^ This Integrated Cell Manipulation Platform (ICMP) coupled with a high-resolution mass spectrometer was further used for quantitative analysis of intracellular metabolites from patient-derived suspension cells such as those in urine from bladder cancer patients (as illustrated in Figure 4 and detailed in section Single-Probe SCMS in Quantitative Studies).^34^ This system not only expanded the range of cell types that could be analyzed with minimal sample preparation but also enhanced specificity and sensitivity in distinguishing cellular features. The versatility of this approach highlighted its potential for personalized medicine, offering a rapid, real-time method to analyze live patient cells and tailor therapeutic strategies.

The semiquantitative applications of the Single-probe SCMS methods have been extended to studying drug-resistant cancer cells. The colorectal cancer cells with irinotecan resistance^43^ possess elevated unsaturated lipids and cancer stem cell markers, pointing to the upregulation of SCD1 as a key factor in resistance. These findings suggested that inhibiting SCD1 could enhance irinotecan sensitivity, offering a potential approach to overcoming drug resistance in clinical treatment. More recently, Chen et al. applied SCMS to evaluate the synergistic effects of combining irinotecan with metformin, an antidiabetic medicine, in irinotecan-resistant colorectal cancer cells.^46^ The study revealed that metformin could downregulate lipids and fatty acids, suppressing cancer cell metabolism. Combining metformin with irinotecan further enhanced the suppression of glycosylated ceramide production, a critical component of cancer cell metabolism. These studies demonstrated the utility of SCMS in investigating drug resistance mechanisms and underscored its potential for broader applications in cancer therapy.

**Figure 2:**
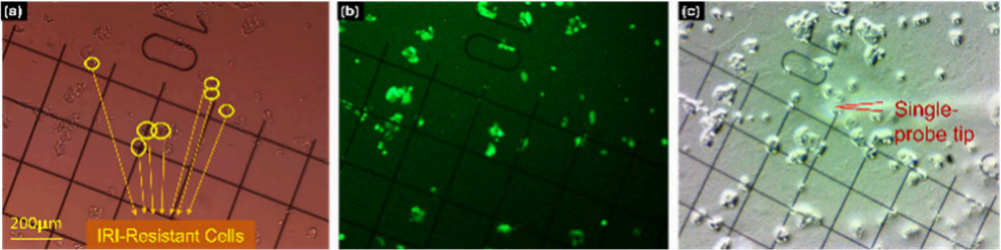
Coupling the Single-probe SCMS with fluorescence microscopy to study cell–cell interactions in a direct co-culture system. Coordinates of single cells in each group were determined by comparing (A) bright-field and (B) fluorescence images of the same coverslip. (C) Metabolites in single cells were measured using the Single-probe SCMS technique. [Reproduced with permission from ref (35). Copyright 2022, Royal Society of Chemistry, London.]

The Single-probe SCMS has coupled with fluorescence microscopy to investigate cell–cell interactions. Chen et al. employed the technique in a co-culture system, which included drug-resistant and drug-sensitive cancer cells, to study metabolism affected by cell–cell interactions^35^ (Figures 2a–c). Two types of co-culture systems were studied, including indirect (two different types of cells were cultured in the same well but separated by Transwell) and direct (two different types of cells were directly cultured in the same well without separation) co-culture systems. In the direct co-culture experiments, one type of cells was labeled with GFP (green fluorescence protein), and fluorescence microscopy was combined with the Single-probe SCMS to analyze metabolites of single cells in each group. The study revealed that drug-sensitive cells exhibited increased resistance and altered metabolic profiles when co-cultured with drug-resistant cells, shedding light on the role of cellular communication in the development of chemotherapy resistance. This application demonstrated the integration of SCMS, and microscopy techniques could provide unique insights into the metabolic shifts driven by cell–cell interactions, paving the way for future studies on the metabolic responses of heterogeneous cell populations.

The Single-probe SCMS has also been coupled with bright-field microscopy to study cell heterogeneity. Nguyen et al. extended the application of SCMS to infectious diseases by investigating host cell heterogeneity during *Trypanosoma cruzi* (*T. cruzi*) infection, the causative agent of Chagas disease (CD).^38^ The study revealed significant metabolic differences between infected cells, which contain stained parasites, and uninfected cells as well as the presence of bystander effect, which indicates uninfected cells adjacent to infected ones displaying altered metabolism. The bystander effect suggested a novel mechanism for lesion development in parasite-free areas, offering crucial insights into the pathogenesis of CD. This work represents the first use of SCMS in studying mammalian-infectious diseases, showcasing the technique’s broad applicability beyond cancer research.

The Single-probe SCMS technique has significantly advanced semiquantitative single-cell metabolomics by enabling precise, real-time analysis of individual cells across diverse biological systems. Its applications cover multiple areas such as marine microorganisms, human diseases, and cell–cell communication, offering unprecedented insight into cellular heterogeneity and metabolic dynamics. As the technique continues to evolve, it holds immense potential for furthering our understanding of complex biological processes and driving innovations in personalized medicine.

#### Single-Probe SCMS in Quantitative Studies

The Single-probe SCMS technique has been used for quantification of anticancer drugs (both amounts and concentrations) in live individual cells under ambient conditions.^21^ Due to its unique design, the internal standard can be added into the sampling solvent (e.g., acetonitrile) at a known concentration. The internal standard can be an isotopically labeled compound or species with the structure highly similar to the target compound.^18,26,106^ When performing quantitative SCMS measurements of drug-treated cells, the Single-probe tip is inserted into a single living cell to extract intracellular chemicals (including drug molecules). Both the internal standard and drug molecules are simultaneously delivered to the nano-ESI emitter for ionization and detected by MS. Multiple factors (e.g., the ion intensities of the drug and internal standard, internal standard’s concentration and flow rate, and data acquisition time) must be considered for the quantification. If the isotopically labeled analogue is not available, the internal standard can be selected from the species with a structure similar to the target compound, whereas a calibration curve must be established. The quantitative SCMS technique makes it possible to accurately estimate the amounts of drugs in individual cells, offering insights into how individual cells metabolize and retain therapeutic agents.

**Figure 3:**
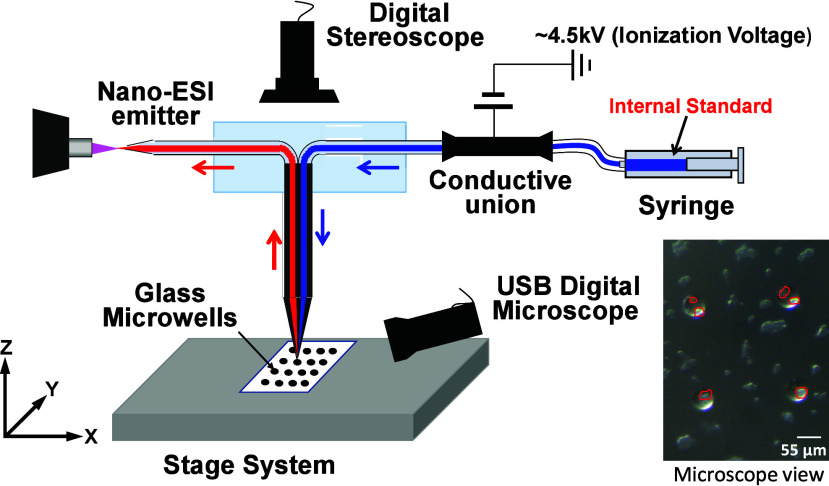
Quantitative Single-probe SCMS experimental setup with individual cells and microwells on glass chips shown in the microscopic image. [Reproduced from ref (39). Copyright 2019, American Chemical Society, Washington, DC.]

This method was first employed to rapidly quantify the absolute amounts of the anticancer drug in individual adherent cancer cells under ambient conditions.^39^ Pan et al. performed the measurement of anticancer drug amounts within live cells (Figure 3). In this study, both HCT-116 and HeLa cell lines were employed to investigate the intracellular uptake of irinotecan under various treatment durations and concentrations. To minimize the diffusion loss of cellular contents and internal standard (irinotecan-d10), glass chips containing microwells (diameter, 55 μm; depth, 25 μm) were used during cell incubation and treatment. Single cells in individual microwells were selected for measurements. The amount of irinotecan within single cells was heterogenous across different cells. When comparing these single cell results with those average values obtained through traditional LC/MS techniques, it was found that the LC/MS approach yielded lower intracellular drug levels. This discrepancy was attributed to drug losses during the sample preparation process in LC/MS, highlighting the advantage of single-cell mass spectrometry in preserving and detecting accurate drug concentrations within cells. This method offers a more direct and precise approach to understanding drug uptake dynamics.

**Figure 4:**
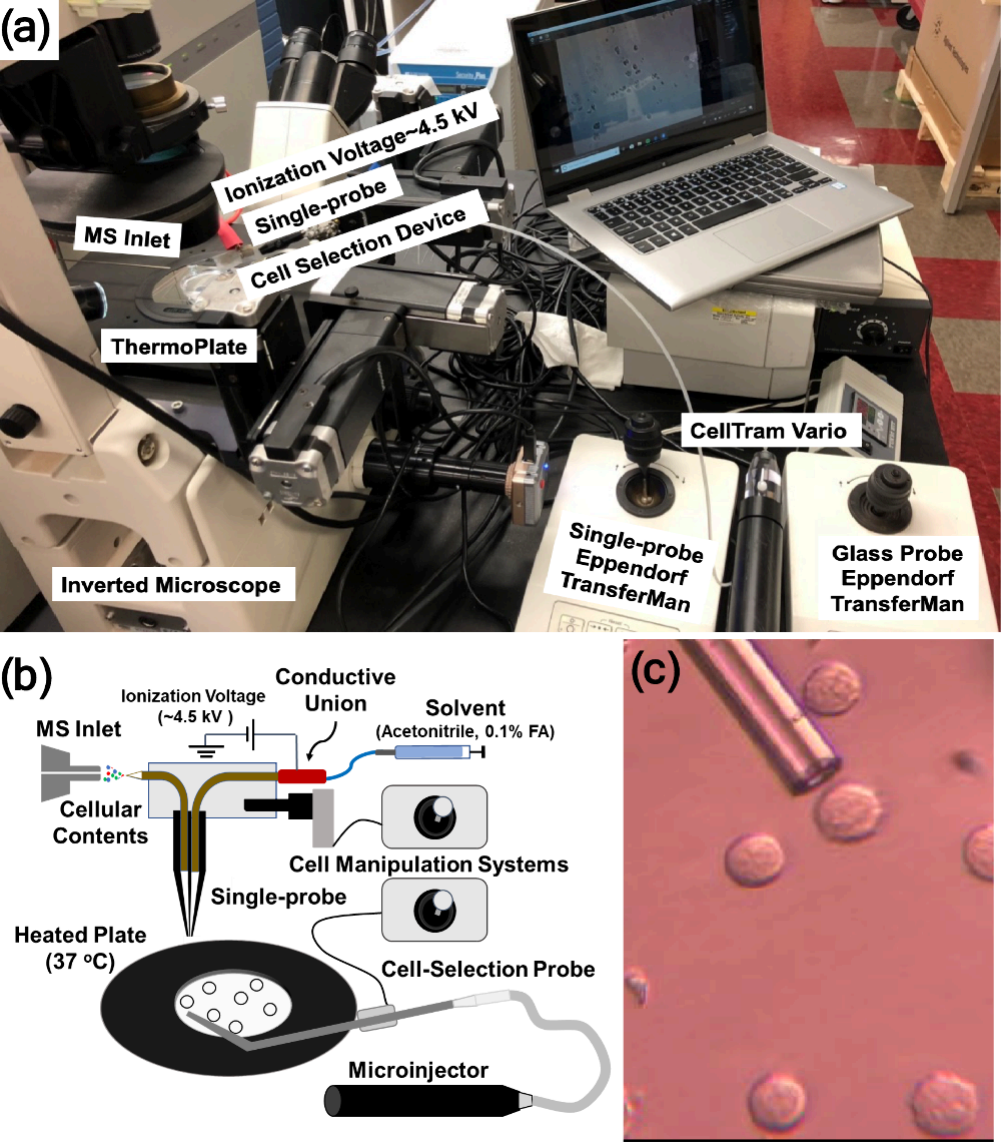
Experimental setup for MS measurement of suspension single cells. (a) The integrated cell manipulation platform (ICMP) coupled with a mass spectrometer. (b) Schematic for analysis of suspended single cells. (c) Microscopic view of K562 cells to be selected using the cell-selection probe. [Reproduced from ref (34). Copyright 2019, American Chemical Society, Washington, DC.]

Recent advancements have integrated the Single-probe with a cell manipulation system, enabling analysis of suspension cells and patient-isolated cells from body fluids (Figures 4a–c).^34^ To extend quantitative SCMS techniques to suspended cells, the Single-probe system was coupled with an integrated cell manipulation platform (ICMP), which consists of an Eppendorf TransferMan cell micromanipulation system, a Nikon Eclipse TE300 inverted microscope, and a Tokai Hit ThermoPlate system. A single cell was selected by the cell selection probe, and the cell diameter was measured using the inverted microscope. In fact, the microscope enables the discrimination between cancerous and noncancerous cells based on their morphological characteristics. The cell was then transferred to the Single-probe tip, where the cell was immediately lysed when contacting the solvent (e.g., acetonitrile containing the internal standard). The single cell lysate and the internal standard were simultaneously detected by MS. Bensen et al. accurately measured intracellular amounts and concentrations of the chemotherapy drug gemcitabine in individual bladder cancer cells, including both K562 cell lines and bladder cancer cells isolated from patients undergoing chemotherapy.^39^ Comparisons with traditional LC/MS results of K562 cells yielded comparable intracellular drug concentrations. This study demonstrates the system’s capacity for real-time, precise quantification of anticancer drug levels in single cells, highlighting its potential for improving personalized chemotherapy regimens.

#### Combing the Single-Probe SCMS with Chemical Reactions

##### Reaction through Noncovalent Interactions

**Figure 5:**
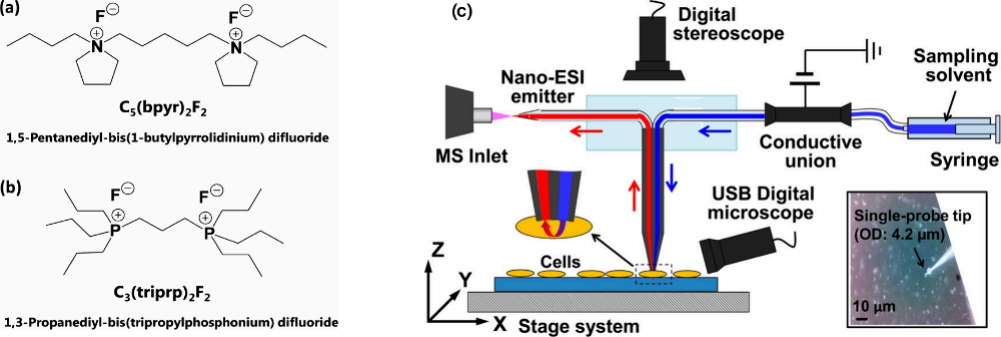
Using dicationic reagents in SCMS studies. Molecular structures of dicationic compounds (a) C5(bpyr)2F2 and (b) C3(triprp)2F2. (c) Schematic drawing of the Single-probe SCMS setup. The inset indicates the insertion of a Single-probe tip into a cell. [Reproduced from ref (33). Copyright 2016, American Chemical Society, Washington, DC.]

In the quest to improve the detection coverage of ionizable cellular metabolites, experiments are often conducted in both positive and negative ionization modes. However, this is particularly challenging in SCMS, due to the extremely limited cellular content available (∼1 pL/cell),^5^ which makes repeated analyses impractical. Addressing this limitation, in 2016, Pan, Rao, and co-workers introduced a unique MS method that facilitates the detection of negatively charged species in single cells using positive ionization mode.^33^ This approach leverages dicationic ion-pairing reagents in conjunction with the Single-probe for real-time reactive SCMS experiments. In their studies, two dicationic compounds, 1,5-pentanediyl-bis(1-butylpyrrolidinium) difluoride (C_5_(bpyr)_2_F_2_) and 1,3-propanediyl-bis(tripropylphosphonium) difluoride (C_3_(triprp)_2_F_2_), were added into the sampling solvent and introduced into single cells (Figures 5a and 5b). These dicationic reagents (2+) formed stable ion pairs with negatively charged (1−) cellular metabolites, transforming them into positively charged (1+) adducts, thus enabling their detection in positive ionization mode with enhanced sensitivity. In three separate SCMS experiments, 192 and 70 negatively charged metabolites were detected as adducts with C_5_(bpyr)_2_F_2_ and C_3_(triprp)_2_F_2_, respectively, along with the detection of other positively charged metabolites, highlighting the capability of this approach to detect a broad spectrum of metabolites. A key advantage of these dicationic compounds lies in their selectivity for complex formation, allowing the discrimination of low-abundance ions with nearly identical *m*/*z* values. Additionally, MS/MS was employed for molecular identification of selected adduct ions. This reactive SCMS method represents a significant advancement by enabling the simultaneous detection of negatively and positively charged metabolites in a single experiment. Most notably, many of the negatively charged metabolites identified using dicationic reagents were undetectable in negative ionization mode alone, demonstrating the enhanced sensitivity offered by this technique. Future studies could explore other compounds to further refine the sensitivity and scope of metabolite detection in single-cell analysis.

##### Reaction through Covalent Interactions

**Figure 6:**
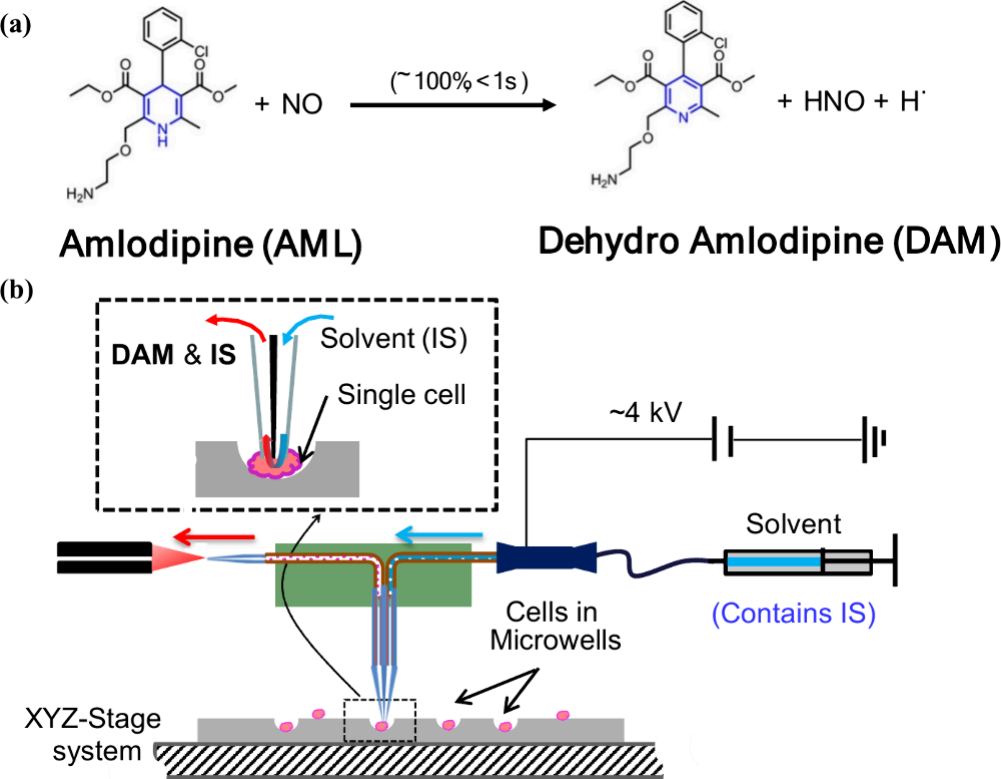
Quantification of NO in single cells. (a) Reaction of AML and NO producing DAM. (b) Using the quantitative Single-probe SCMS cell setup to quantify NO in single cells. A glass chip containing microwells is used for cell culture and the SCMS experiment. [Reproduced from ref (36). Copyright 2023, American Chemical Society, Washington, DC.]

Lan et al. introduced a novel method for indirect quantifying intracellular nitric oxide (NO) by means of chemical reactions at the single-cell level (Figures 6a and 6b).^36^ NO, a reactive and short-lived molecule (with a half-life of less than one second), plays a critical role in various biological processes, including angiogenesis in tumors. Quantifying NO at the single-cell level remains challenging due to the small size of cells and NO’s reactive nature. There are two main pathways for NO production: exogenous (provided by NO donor compounds) and endogenous (produced by cells). Clinically, NO donors are used in the treatment of conditions such as high blood pressure and heart disease. Additionally, the anticancer drug doxorubicin (DOX) can increase endogenous NO levels via the catalytic activity of nitric oxide synthase (NOSs). Given NO’s crucial biological functions, developing a method to accurately quantify NO at the single-cell level is highly significant. Lan et al. proposed a method based on a quantitative reaction between NO and amlodipine (AML), a compound containing the Hantzsch ester group. The reaction between NO and AML yields dehydroamlodipine (DAM), which can then be detected and quantified using the Single-probe SCMS technique. Importantly, AML reacts selectively with NO, exhibiting 100% efficiency without interference from other reactive species within the cell. In their study, individual cells were adhered to glass chips containing microwells and were subsequently treated with AML under different experimental conditions. To induce NO production, two compounds were used: sodium nitroprusside (SNP) (to generate exogenous NO) and doxorubicin (DOX) (to stimulate production of endogenous NO). The Single-probe SCMS system was employed for NO quantification, with acetonitrile (ACN) containing 0.1% formic acid (FA) and 1.0 μM OXF (internal standard) used as the sampling solution. Results from the SCMS studies demonstrated that intracellular NO levels exhibited heterogeneous distributions across the treated cells under all experimental conditions. This method provides a robust approach to quantifying NO at the single-cell level, offering insights into the complex biological roles of NO in cellular systems.

#### Improving Cell Sample Preparation for Robust Single-Probe SCMS Analysis

**Figure 7:**
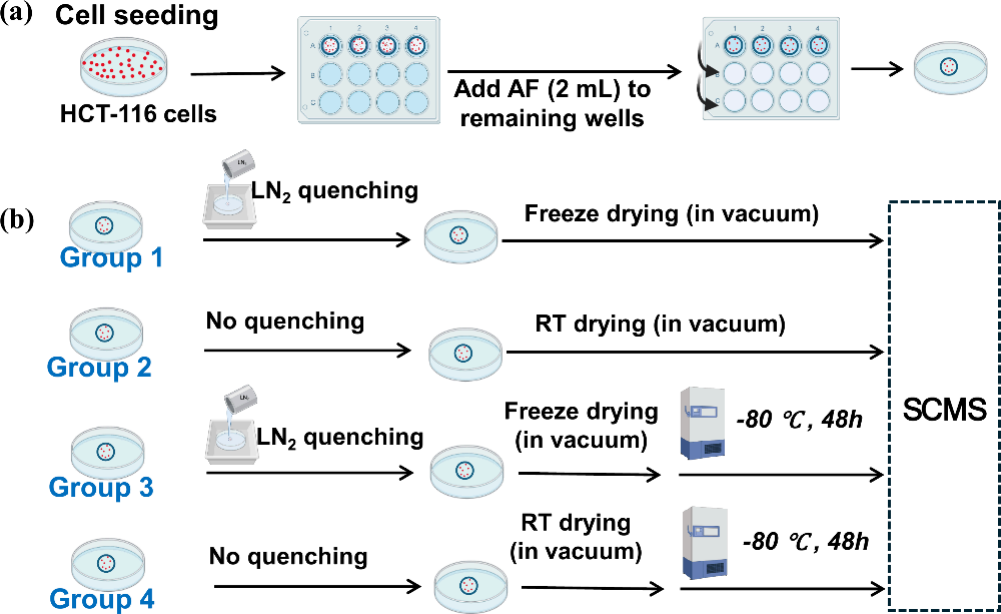
Overall workflow of SCMS studies of the impact of LN_2_ quenching and −80 °C storage (48 h) on metabolites’ profiles in single cells. (a) Cell seeding and washing by AF solution. (b) Four groups of cells were used in experiments: Group 1, cells that were washed, quenched, and freeze-dried (no storage); Group 2, cells that were washed and dried at (room temperature) RT (no quenching and storage); Group 3, cells that were quenched, freeze-dried, and stored; Group 4, cells were dried at RT and stored (no quenching). [Reproduced from ref (107). Copyright 2024, ChemrXiv.]

Maintaining the metabolic integrity of live cells during sample transport, storage, or extended measurements is critical, particularly given the rapid turnover rate of metabolites and low throughput of most ambient-based SCMS techniques, which require substantial time to manually select and analyze a statistically significant number of cells. A recent study developed a robust methodology to preserve cellular metabolomic profiles for SCMS experiments, addressing the challenge in most ambient SCMS metabolomics studies (Figure 7).^107^ This study introduced a cell preparation protocol combining washing by volatile salt (ammonium formate (AF)) solution, rapid quenching in liquid nitrogen (LN_2_), vacuum freeze-drying, and storage at −80 °C to stabilize cell metabolites for SCMS analysis. Experimental findings demonstrated that LN_2_ quenching effectively preserved the overall metabolome, while storage at −80 °C for 48 h caused minor changes in metabolite profiles of quenched cells. In contrast, unquenched cells exhibited significant metabolic alterations despite low-temperature storage. Further investigation revealed the necessity of quenching to maintain metabolic integrity and emphasized minimizing low-temperature storage duration to limit metabolic perturbations. The proposed method is readily applicable to SCMS workflows, ensuring metabolite stability during extended studies while maintaining the fidelity of metabolic profiles.

#### Combining Advanced Data Analysis Methodologies with Single-Probe SCMS Experiments

The integration of SCMS methods with innovative data analysis techniques has significantly advanced the field of single-cell metabolomics. A variety of data processing and analysis methods have been employed to extract meaningful insights from the complex data generated from the Single-probe SCMS experiments, extending the applications of these techniques.^32,38,51,108^

##### SCMS Data Pretreatment

Liu et al. reported a generalized data analysis workflow to pretreat the Single-probe SCMS data.^37^ This data preprocessing workflow includes multiple key steps for data refinement: (1) removal of exogenous ion signals originated from culture medium and sampling solvent; (2) filtering instrument noise, which typically comprises 20%–40% of detected peaks, through low-intensity ion exclusion; and (3) normalization of metabolite ion intensities to the total ion count. These steps were shown to effectively reduce data dimensionality while retaining crucial metabolite information, though challenges remain in distinguishing true metabolite signals from low-abundance noise.^51^ This generalized data analysis workflow can be seamlessly integrated with raw datasets, enabling thorough metabolomic analyses across different experimental conditions.

The introduction of MassLite by Zhu et al. marks a notable advancement in the pretreatment of metabolomics data. This software package is an integrated Python platform with a user-friendly graphical interface for processing data in standard .mzML format. This tool is suitable to handle data from intermittent acquisition processes, enabling efficient segmentation of ion signals from individual cells. MassLite also retains low-intensity metabolite signals within complex single-cell data, broadening the scope of detectable molecular species from limited analyte content. Additionally, this tool incorporates functions for void scan filtering, dynamic grouping, and advanced background removal, all of which enhance data quality and processing efficiency. Furthermore, MassLite automates cell region selection, replacing the manual process to enhance processing throughput. Overall, MassLite serves as a vital tool for advancing SCMS research, streamlining data preprocessing, and facilitating more accurate metabolomic analyses.

##### SCMS Data Analysis by Machine Learning

While significant progress has been made in understanding drug resistance mechanisms, predicting a drug-resistant phenotype before starting chemotherapy remains underexplored, potentially resulting in ineffective treatments and unwanted toxicity for patients. For the first time, the integration of the Single-probe SCMS with machine learning techniques was performed by Liu et al. to quickly and accurately predict the phenotypes of unknown single cells. This innovative approach, facilitated by the Single-probe, offers a solution for the rapid and reliable prediction of drug-resistant cancer cell phenotypes such as those associated with chemoresistance mechanisms (e.g., cell adhesion-mediated drug resistance (CAM-DR)).^45^ Advanced data analysis, incorporating machine learning algorithms, was subsequently used to process complex metabolomic data. Specifically, random forest (RF), penalized logistic regression (LR), and artificial neural networks (ANNs) were used for analyzing pretreated single-cell metabolomic datasets. By integrating a diverse range of cellular metabolites, these models achieved significantly improved predictive accuracy (*p*-value < 0.05) compared to other approaches that relied solely on metabolic biomarkers identified through two-sample *t*-tests or PCA loading plots. This highlights the effectiveness of our methodology in enhancing model performance.

Yao et al.^109^ developed MetaPhenotype, a meta-learning-based model designed to address limitations in adaptability and transferability often encountered in machine learning models for SCMS data analysis. SCMS is a powerful tool for investigating cellular heterogeneity, such as phenotypes, through the variation of molecular species in individual cells. However, its application to rare cell populations is often constrained by the limited availability of cell samples. To overcome these challenges, two pairs of isogenic melanoma cancer cell lines (each has primary and metastatic phenotypes) were analyzed using the Single-probe SCMS technique. Both control and drug-treated cells were analyzed. The SCMS metabolomics data of one cell pair (no drug treatment) served as the training and evaluation datasets for MetaPhenotype, which was subsequently applied to classify the remaining data. The MetaPhenotype model demonstrated rapid adaptation and exceptional transferability, achieving high prediction accuracy of over 90% with minimal new training samples. Moreover, it enabled the identification of a small subset of critical molecular species essential for phenotype classification. This work highlights the potential of MetaPhenotype to lower the demand for extensive sample acquisition, facilitating accurate cell phenotype classification even with limited SCMS datasets. The applicability of MetaPhenotype extends beyond melanoma cell lines and the specific SCMS platform employed in this study, offering potential for broader use in metabolomics studies across diverse SCMS platforms and cell systems.

##### SCMS Data Analysis by Biostatistics

A notable study by Liu et al. illustrates the application of the Single-probe SCMS experiment combined with SinCHet-MS (Single Cell Heterogeneity for Mass Spectrometry) software to investigate tumor cell heterogeneity and cellular subpopulations.^32^ They analyzed the metabolomic profiles of drug-sensitive and drug-resistant melanoma cells (WM115 and WM266-4) treated with vemurafenib. The data were subjected to batch effect correction, subpopulation analysis, and biomarker prioritization. Notably, the findings showed that drug-sensitive cells developed a new subpopulation after treatment, while drug-resistant cells only showed changes in existing subpopulation proportions. There are a few highlights of this work. First, for the first time, effect correction in SCMS studies was performed using SinCHet-MS. Second, the subpopulations of cells can be quantified using this bioinformatics tool. Third, new algorithms used in this software allow for prioritizing biomarkers of subpopulations of cells.

These contributions underscore the transformative impact of combining Single-probe SCMS experiments with sophisticated data analysis techniques, paving the way for improved understanding of cellular behaviors and therapeutic responses.

### Single-Probe MS Imaging (MSI)

**Figure 8:**
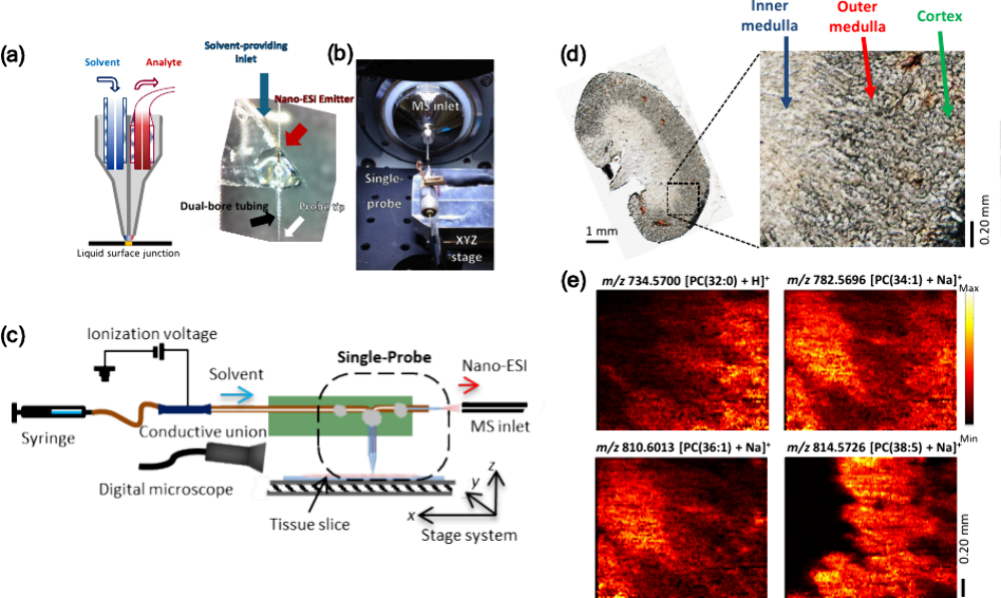
Single-probe MSI of tissue slices. Photographs of (a) the Single-probe and (b) setup of the Single-probe during MSI measurement. (c) Schematic of the Single-probe MSI system. (d) Optical image of the mouse kidney section showing the region of MSI measurement. (e) MS images of selected metabolites (8.5 μm × 20 μm pixel size). [Reproduced from ref (110). Copyright 2015, American Chemical Society, Washington, DC.]

As a microscale sampling and ionization device, the Single-probe can be coupled to MS for other studies. The Single-probe MS imaging (MSI) technique, first introduced in 2015, is a novel tool for analyzing biomolecules on tissue slices with high spatial resolution under ambient conditions.^110−112^ During the MSI experiment, the Single-probe tip is placed closely above the tissue slice, and the solvent junction at the tip performs in-situ surface microextraction, and the extracted molecules are immediately analyzed by MS (Figures 8a–c). Using programmed stage control system, the Single-probe tip performs continuous raster sampling of the region of interest in tissue. MS images of ions of interest can be constructed using a visualization tool. The Single-probe is capable of producing MSI images of biological tissue slices with a spatial resolution as fine as 8.5 μm (Figures 8d and 8e), making it one of the highest resolutions among ambient MSI methods available.

#### Combining the Single-Probe MSI with Chemical Reactions

Due to its unique design, the sampling solvent of the Single-probe can be flexibly selected. Similar to the relevant application in SCMS studies,^33^ the use of dicationic compounds (i.e., [C_5_(bpyr)_2_F_2_] and [C_3_(triprp)_2_F_2_]) (Figure 5) in MSI experiments enabled the detection of negatively charged species in the positive ion mode.^26^ Particularly, detection of metabolites in the range of 600–900 *m*/*z* was improved with enhanced ion intensities compared to regular negative ionization modes. This technique also allowed the detection of metabolites that were previously undetectable under standard conditions.

#### Combining Advanced Data Analysis with Single-Probe MSI Experiments

Due to their high dimensionality, high complexity, and large size, extracting essential biological information from MSI data is generally challenging. To facilitate the relevant studies, advanced data analysis methods have been developed and combined with the Single-probe MSI experiments.^23,24,27,112^

Tian et al.^24^ developed a data analysis method using Multivariant Curve Resolution (MCR) and Machine Learning (ML) approaches, and then used it to analyze the MSI data from a mouse kidney slice. This method involved four main steps: data preprocessing, MCR-Alternating Least Squares (ALS), supervised ML (e.g., Random Forest), and unsupervised ML (e.g., Clustering Large Applications (CLARA) and Density-based Spatial Clustering of Applications with Noise (DBSCAN)). A key step was using t-SNE, a dimensionality reduction tool, to process and visualize the complex datasets. For supervised ML methods, predefined histological regions identified through MCR-ALS were used to train the models. In unsupervised methods, t-SNE prepared the data for clustering. The combination of these approaches provided a more thorough understanding of chemical and spatial features in the data. Other machine learning methods were then developed to improve the MSI data analysis. In a study involving slices of cancer spheroids, the Single-probe was used to examine the effects of the anticancer drug Irinotecan on colorectal cancer (HCT-116) spheroids.^23^ By obtaining spatially resolved metabolomic profiles, the technique revealed how the drug affected the abundance of metabolites in different regions of the 3D tumor model. ML techniques, such as Random Forest and CLARA, were employed to analyze the MSI data, improving the identification and classification of metabolomic features.

**Figure 9:**
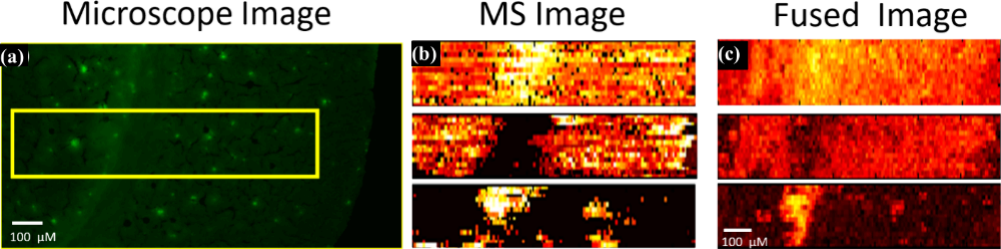
Fusion of fluorescence microscopy image and MS image. (a) Fluorescence microscopy image of a 5XFAD mouse brain slice stained using Thioflavin S. (b) Original MS images of metabolites ([PC(34:1) + H]^+^ (*m*/*z* 760.5851) (top), [PC(38:6) + H]^+^ (*m*/*z* 844.5218) (middle), and [LPC(18:0) + H]^+^ (*m*/*z* 524.3693) (bottom)) and (c) their fused images. All metabolites were identified using MS^2^ from the tissue slice, and the results were compared with METLIN. [Reproduced from ref (27). Copyright 2019, American Chemical Society, Washington, DC.]

The MS images obtained from the Single-probe MSI experiments can be integrated with fluorescence microscopy images through image fusion (Figure 9a–c). In Alzheimer’s disease (AD) research, the Single-probe was used to investigate the spatial distribution of metabolites around amyloid-beta (Aβ) plaques in an AD mouse brain.^27^ Image fusion allowed researchers to correlate histological markers (detected through fluorescence microscopy) with metabolomic features (observed through MSI). This combined approach improved spatial resolution (∼5 μm) and provided insights into abnormal metabolite expressions, such as lysophospholipids, malic acid, and glutamine, that are linked to the progression of AD.

### Single-Probe Mass Spectrometry in Live Multicellular Tumor Spheroids

**Figure 10:**
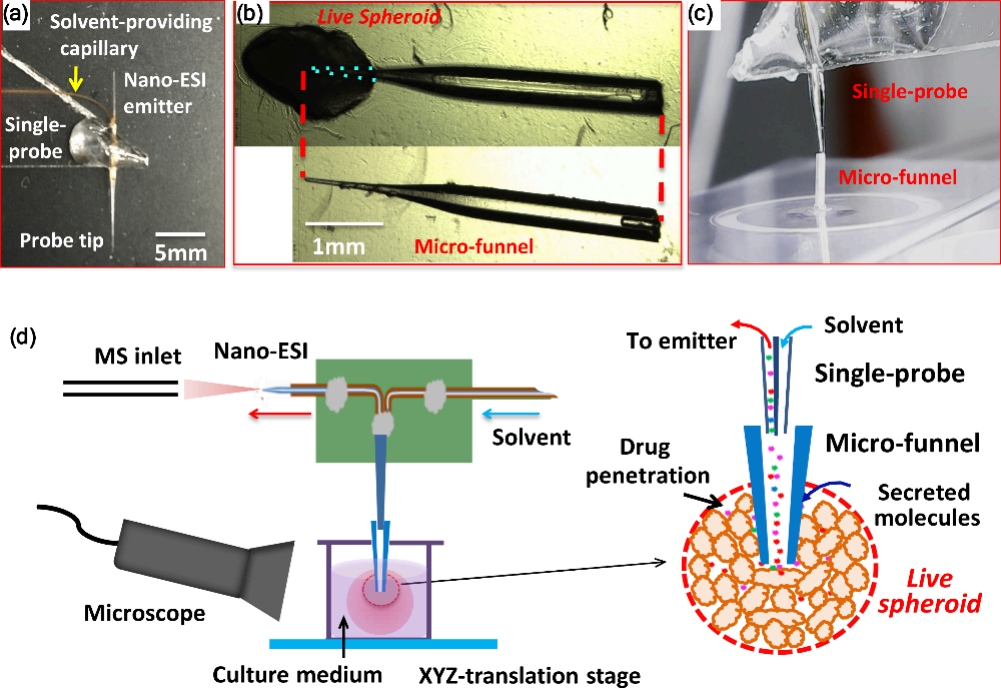
Combining the microfunnel and Single-probe technique for MS analysis of extracellular compounds in live spheroids. (a) Photograph of a Single-probe device. (b) Photograph of a microfunnel before and after being implanted into a spheroid. (c) Coupled microfunnel and Single-probe device. (d) Real-time MS analysis of extracellular compounds in a live spheroid using the coupled microfunnel and Single-probe device. [Reproduced from ref (28). Copyright 2017, American Chemical Society, Washington, DC].

The Single-probe can be used as a microscale sampling device to extract analytes for direct MS analysis. In a study by Sun et al., the integration of the microfunnel, which was implanted into a spheroid, with the Single-probe provided an innovative approach to analyze extracellular metabolites in live multicellular tumor spheroids (Figure 10). This work focused on understanding the effects of anticancer drug treatments in the tumor microenvironment.^28^ This technique is particularly valuable for capturing undiluted extracellular compounds inside single spheroids, a critical area due to its unique microenvironment and potential for harboring drug-resistant cells. To carry out this work, the researchers first developed the microfunnel from a biocompatible fused silica capillary with a fine tip (∼25 μm), enabling precise implantation into the spheroid to collect extracellular compounds. The spheroids, cultured using a colon carcinoma cell line (HCT-116), were treated with the anticancer drug irinotecan under various concentrations and durations. The microfunnel allowed for targeted sampling, accumulating metabolites in a microscale environment that would otherwise be challenging to access without dilution or selection bias. Once metabolites were collected, the Single-probe was inserted into the opening of the microfunnel to extract these metabolites and was analyzed by MS. The changes in the spheroid’s extracellular lipid profile were observed, particularly in phospholipids and glycerides, with increased lipid abundance as drug treatment concentration and exposure time increased. These results indicated that irinotecan prompted significant shifts in lipid metabolites, which could contribute to drug-resistance mechanisms within central tumor cells. This study’s workflow demonstrates an effective methodology for profiling the extracellular environment of live spheroids, making it a valuable tool for investigating drug response, cellular communication, and resistance mechanisms in three-dimensional (3D) cancer models.

## STUDIES PERFORMED USING OTHER SINGLE-PROBE-BASED DESIGNS

The general design of the Single-probe device has been adopted and modified by other researchers for a variety of different studies.

### Quantitative MSI Studies

In 2021, Wu et al. applied the Single-probe technique for per-pixel absolute quantification of endogenous lipidomes through model prediction of mass-transfer kinetics.^113^ This method enabled ambient liquid extraction MSI in rat cerebellum, utilizing phosphatidylcholine (PC) and cerebroside (CB) standards doped in the extraction solvent. By studying the extraction kinetics of endogenous lipids during the probe’s stationary phase in each tissue pixel, the team could gather detailed kinetic data.

### Enrichment of Low-Abundance Analytes on Biological Tissue Slices

In 2021, Wang et al. further leveraged the Single-probe fabrication to create a microprobe with a larger tip size, suitable for ambient liquid extraction MSI but not single-cell analysis.^114^ This study aimed to address the limited imaging coverage of low-abundance or low-polarity lipids, such as glycerolipids and sphingolipids, in complex tissues. To do so, they applied a porous graphitic carbon (PGC) material to imprint brain tissue sections selectively, enriching neutral lipids while removing polar phospholipids. Subsequent scanning of the PGC-imprinted tissue with the ambient liquid extraction MSI system revealed that hydrophobic interactions dominate in protic solvents on the PGC surface, while polar interactions dominate in aprotic solvents. A recent study performed by this group presents a novel MSI approach that enhances spatial lipidomics analysis using a graphene oxide/titanium dioxide (GO/TiO_2_) nanocomposite as a mixed-mode adsorptive material.^115^ By combining the chelation affinity of TiO_2_ with the hydrophobic interaction of GO, the material facilitates selective enrichment of poorly ionizable glycolipids and glycerides while reducing ion suppression and peak interference from high-abundance polar lipids. Optimized solvent systems enabled on-plate separation of lipid classes and efficient two-step ambient liquid extraction MSI. This method significantly improved lipid coverage, detecting a greater variety of glycolipids, glycerides, and phospholipids compared to traditional MSI techniques. Application to rat cerebellum tissue demonstrated higher imaging quality and comprehensive lipid profiling, advancing the depth and scope of spatial lipidomics studies. Their future work will focus on scaling the nanocomposite coating for single-cell MSI.

In 2024, Wu et al. advanced the Single-probe for ambient liquid extraction MSI studies aimed at enhancing the detection of poorly ionizable lipids in brain tissue using a Lewis acidic metal–organic framework (MOF).^116^ In this study, the sample was placed on a triaxial platform, with the Single-probe affixed in a perpendicular orientation, relative to the sample surface. The team employed 1% FA-MeOH as the extraction solvent at a flow rate of 5 μL min^–1^, delivered via a syringe pump, while a vacuum pump drew the solvent into the probe, creating a stable liquid junction with a precise 10 μm distance between the probe tip and sample surface. This approach effectively mitigated ion suppression by phospholipids in MSI, significantly improving the detection coverage of low-abundance, poorly ionizable lipids.

### Using Chemical Reactions to Improve the Detection of Low-Abundance Analytes with Low Ionization Efficiencies

In 2024, Lu et al. developed a novel method to address challenges in lipidomics, specifically for glycosphingolipids (GSLs), which are difficult to ionize and analyze.^117^ This method introduces a photoinduced enrichment and deglycosylation approach, implemented in an ambient liquid extraction MS system, to improve GSL detection coverage and structural elucidation in single-cell analysis. Using TiO_2_ in ammonia-based protic solvents, GSL standards were selectively adsorbed. Under UV irradiation, GSLs underwent deglycosylation (losing one hexosyl group) with a high conversion efficiency (>70%), then desorbed from TiO_2_. Coating the TiO_2_ onto a capillary probe enabled selective GSL enrichment while separating them from high-abundance phospholipids, reducing ion suppression. UV exposure triggered rapid photodesorption without solvent changes, achieving 6-fold GSL enrichment. This enhanced GSL detection 9-fold, compared to traditional methods, allowing for detailed fatty acyl and sphingosine chain elucidation through increased MS/MS fragmentation. The method was applied to lipidomics in nerve cells, identifying 31 lipids, including 11 GSLs, and detecting alterations in five hexosylceramides after neuron injury. This innovative TiO_2_-coated probe demonstrated low limits of detection (3.7 ng/mL), high linearity (*r* > 0.99), and repeatability (RSD < 20%). In brain tissue analysis, this technique identified 38 more lipids than using conventional methods. Overall, this approach significantly advances single cell lipidomics by enhancing GSL detection and structural analysis, providing valuable insights for biomedical and photo-oxidation research.

## FUTURE ASPECTS

Since it was first introduced in 2014, the Single-probe-based methods have demonstrated their capabilities in various studies of microscale bioanalyses, such as single cells, tissue slices, and 3D tumor models, in ambient conditions. Implemented with other techniques in instrumentation (e.g., microscopy and precise manipulation), chemical reactions, and surface functionalization, applications of these methods have been largely extended. The advancement in data analysis tools (e.g., multivariate analysis and machine learning) enables extraction of essential information from complex data. Regardless of their advantages, broad applications of the Single-probe-based methods still face multiple challenges. In Single-probe SCMS studies, cell sampling must be manually performed using the XYZ-stage system guided by a microscope. Although this is beneficial for studies of target cells, which can be labeled by dyes or fluorescent proteins, among heterogeneous populations, these manual procedures largely limit the analysis throughput. In fact, microfluidics techniques have been implemented to SCMS metabolomics studies.^118−122^ Similar strategies can be potentially adopted by the Single-probe SCMS setup to improve its analysis throughput. In Single-probe MSI studies, maintaining the robustness of the experimental setup for stable data acquisition (e.g., several hours) has been challenging. These issues can be mitigated by fabricating robust probes with carefully adjusted tip sizes and shapes. In fact, taking advantage of modern microfabrication techniques (e.g., micromachining, microinjection, and 3D printing), the fabrication of high-quality Single-probe devices can be automated and standardized, promoting their widespread adoption with high consistency and reliability across laboratories. In addition, enclosed, environmentally controlled setups can further enhance reproducibility by mitigating external influences such as temperature and humidity variations. In principle, the Single-probe setup can be customized and coupled with any model of mass spectrometer with a suitable interface. Its open design allows for flexible customization of translation stage system, microscope, and solvent and reagent selection and delivery. Similar to all other MS studies, the Single-probe MS techniques can reap the benefit of rapid advancements in modern mass spectrometers (e.g., detection sensitivity, mass resolution, and data acquisition speed). Collectively, these technology innovations and advancements will broaden the utility of Single-probe MS methods, solidifying their roles in advancing cutting-edge biological research.

## References

[ref1] Xin X.; Wang H.; Han L.; Wang M.; Fang H.; Hao Y.; Li J.; Zhang H.; Zheng C.; Shen C. Single-Cell Analysis of the Impact of Host Cell Heterogeneity on Infection with Foot-and-Mouth Disease Virus. J. Virol 2018, 92 ( (9), ). DOI: 10.1128/JVI.00179-18. PMC5899210 29444939

[ref2] Raj A.; Rifkin S. A.; Andersen E.; van Oudenaarden A. Variability in gene expression underlies incomplete penetrance. Nature 2010, 463 (7283), 913–918. 10.1038/nature08781. 20164922 PMC2836165

[ref3] Elowitz M. B.; Levine A. J.; Siggia E. D.; Swain P. S. Stochastic Gene Expression in a Single Cell. Science 2002, 297, 1183 10.1126/science.1070919. 12183631

[ref4] Fritzsch F. S.; Dusny C.; Frick O.; Schmid A. Single-cell analysis in biotechnology, systems biology, and biocatalysis. Annu. Rev. Chem. Biomol Eng. 2012, 3, 129–155. 10.1146/annurev-chembioeng-062011-081056. 22468600

[ref5] Schmid A.; Kortmann H.; Dittrich P. S.; Blank L. M. Chemical and biological single cell analysis. Curr. Opin Biotechnol 2010, 21 (1), 12–20. 10.1016/j.copbio.2010.01.007. 20167469

[ref6] Xu Y.; Wang S.; Feng Q.; Xia J.; Li Y.; Li H. D.; Wang J. scCAD: Cluster decomposition-based anomaly detection for rare cell identification in single-cell expression data. Nat. Commun. 2024, 15 (1), 7561 10.1038/s41467-024-51891-9. 39215003 PMC11364754

[ref7] Nguyen A.; Khoo W. H.; Moran I.; Croucher P. I.; Phan T. G. Single Cell RNA Sequencing of Rare Immune Cell Populations. Front Immunol 2018, 9, 1553 10.3389/fimmu.2018.01553. 30022984 PMC6039576

[ref8] Sun M.; Yang Z. Metabolomic Studies of Live Single Cancer Stem Cells Using Mass Spectrometry. Anal. Chem. 2019, 91 (3), 2384–2391. 10.1021/acs.analchem.8b05166. 30582812 PMC6582952

[ref9] Jiang M.; Xu X.; Guo G. Understanding embryonic development at single-cell resolution. Cell Regen 2021, 10 (1), 10 10.1186/s13619-020-00074-0. 33501559 PMC7838059

[ref10] Klein A. M.; Mazutis L.; Akartuna I.; Tallapragada N.; Veres A.; Li V.; Peshkin L.; Weitz D. A.; Kirschner M. W. Droplet barcoding for single-cell transcriptomics applied to embryonic stem cells. Cell 2015, 161 (5), 1187–1201. 10.1016/j.cell.2015.04.044. 26000487 PMC4441768

[ref11] Nath A.; Bild A. H. Leveraging Single-Cell Approaches in Cancer Precision Medicine. Trends in Cancer 2021, 7 (4), 359–372. 10.1016/j.trecan.2021.01.007. 33563578 PMC7969443

[ref12] Alberter B.; Klein C. A; Polzer B. Single-cell analysis of CTCs with diagnostic precision: opportunities and challenges for personalized medicine. Expert Rev. Mol. DIagnostics 2016, 16 ( (1), ). DOI: 25 10.1586/14737159.2016.1121099. 26567956

[ref13] Beckman R. A.; Schemmann G. S.; Yeang C. H. Impact of genetic dynamics and single-cell heterogeneity on development of nonstandard personalized medicine strategies for cancer. Proc. Natl. Acad. Sci. U. S. A. 2012, 109 (36), 14586–14591. 10.1073/pnas.1203559109. 22891318 PMC3437850

[ref14] Jovic D.; Liang X.; Zeng H.; Lin L.; Xu F.; Luo Y. Single-cell RNA sequencing technologies and applications: A brief overview. Clin Transl Med. 2022, 12 (3), e694 10.1002/ctm2.694. 35352511 PMC8964935

[ref15] Dutta A. K.; Alberge J. B.; Sklavenitis-Pistofidis R.; Lightbody E. D.; Getz G.; Ghobrial I. M. Single-cell profiling of tumour evolution in multiple myeloma - opportunities for precision medicine. Nat. Rev. Clin Oncol 2022, 19 (4), 223–236. 10.1038/s41571-021-00593-y. 35017721

[ref16] Li Y.; Ma L.; Wu D.; Chen G. Advances in bulk and single-cell multi-omics approaches for systems biology and precision medicine. Brief Bioinform 2021, 22 ( (5), ). DOI: 10.1093/bib/bbab024. 33778867

[ref17] Lee S.; Vu H. M.; Lee J. H.; Lim H.; Kim M. S. Advances in Mass Spectrometry-Based Single Cell Analysis. Biology (Basel) 2023, 12 ( (3), ). DOI: 395 10.3390/biology12030395. 36979087 PMC10045136

[ref18] Lan Y.; Zou Z.; Yang Z. Single Cell mass spectrometry: Towards quantification of small molecules in individual cells. TrAC Trends in Analytical Chemistry 2024, 174. DOI: 117657 10.1016/j.trac.2024.117657. 39391010 PMC11465888

[ref19] Qiu S.; Cai Y.; Yao H.; Lin C.; Xie Y.; Tang S.; Zhang A. Small molecule metabolites: discovery of biomarkers and therapeutic targets. Signal Transduct Target Ther 2023, 8 (1), 132 10.1038/s41392-023-01399-3. 36941259 PMC10026263

[ref20] Muthubharathi B. C.; Gowripriya T.; Balamurugan K. Metabolomics: small molecules that matter more. Mol. Omics 2021, 17 (2), 210–229. 10.1039/D0MO00176G. 33598670

[ref21] Pan N.; Rao W.; Kothapalli N. R.; Liu R.; Burgett A. W.; Yang Z. The single-probe: a miniaturized multifunctional device for single cell mass spectrometry analysis. Anal. Chem. 2014, 86 (19), 9376–9380. 10.1021/ac5029038. 25222919

[ref22] Liu R.; Zhang G.; Yang Z. Towards Rapid Prediction of Drug-resistant Cancer Cell Phenotypes- Single Cell Mass Spectrometry Combined with Machine Learning. Chem. Commun. 2019, 55 (5), 616 10.1039/C8CC08296K. PMC6640148 30525135

[ref23] Tian X.; Zhang G.; Zou Z.; Yang Z. Anticancer Drug Affects Metabolomic Profiles in Multicellular Spheroids: Studies Using Mass Spectrometry Imaging Combined with Machine Learning. Anal. Chem. 2019, 91 (9), 5802–5809. 10.1021/acs.analchem.9b00026. 30951294 PMC6573030

[ref24] Tian X.; Zhang G.; Shao Y.; Yang Z. Towards enhanced metabolomic data analysis of mass spectrometry image: Multivariate Curve Resolution and Machine Learning. Anal. Chim. Acta 2018, 1037, 211–219. 10.1016/j.aca.2018.02.031. 30292295 PMC6176743

[ref25] Rao W.; Pan N.; Yang Z. Applications of the Single-probe: Mass Spectrometry Imaging and Single Cell Analysis under Ambient Conditions. J. Vis Exp 2016, ( (112), ). DOI: 10.3791/53911. PMC4924803 27341402

[ref26] Rao W.; Pan N.; Tian X.; Yang Z. High-Resolution Ambient MS Imaging of Negative Ions in Positive Ion Mode: Using Dicationic Reagents with the Single-Probe. J. Am. Soc. Mass Spectrom. 2016, 27 (1), 124–134. 10.1007/s13361-015-1287-7. 26489411 PMC4924531

[ref27] Tian X.; Xie B.; Zou Z.; Jiao Y.; Lin L. E.; Chen C. L.; Hsu C. C.; Peng J.; Yang Z. Multimodal Imaging of Amyloid Plaques: Fusion of the Single-Probe Mass Spectrometry Image and Fluorescence Microscopy Image. Anal. Chem. 2019, 91 (20), 12882–12889. 10.1021/acs.analchem.9b02792. 31536324 PMC6885010

[ref28] Sun M.; Tian X.; Yang Z. Microscale Mass Spectrometry Analysis of Extracellular Metabolites in Live Multicellular Tumor Spheroids. Anal. Chem. 2017, 89 (17), 9069–9076. 10.1021/acs.analchem.7b01746. 28753268 PMC5912160

[ref29] Zhu Y.; Liu R.; Yang Z. Redesigning the T-probe for mass spectrometry analysis of online lysis of non-adherent single cells. Anal. Chim. Acta 2019, 1084, 53–59. 10.1016/j.aca.2019.07.059. 31519234 PMC6746249

[ref30] Liu R.; Pan N.; Zhu Y.; Yang Z. T-Probe: An Integrated Microscale Device for Online In Situ Single Cell Analysis and Metabolic Profiling Using Mass Spectrometry. Anal. Chem. 2018, 90 (18), 11078–11085. 10.1021/acs.analchem.8b02927. 30119596 PMC6583895

[ref31] Zhu Y.; Wang W.; Yang Z. Combining Mass Spectrometry with Paterno-Buchi Reaction to Determine Double-Bond Positions in Lipids at the Single-Cell Level. Anal. Chem. 2020, 92 (16), 11380–11387. 10.1021/acs.analchem.0c02245. 32678580 PMC7482314

[ref32] Liu R.; Li J.; Lan Y.; Nguyen T. D.; Chen Y. A.; Yang Z. Quantifying Cell Heterogeneity and Subpopulations Using Single Cell Metabolomics. Anal. Chem. 2023, 95 (18), 7127–7133. 10.1021/acs.analchem.2c05245. 37115510 PMC11476832

[ref33] Pan N.; Rao W.; Standke S. J.; Yang Z. Using Dicationic Ion-Pairing Compounds To Enhance the Single Cell Mass Spectrometry Analysis Using the Single-Probe: A Microscale Sampling and Ionization Device. Anal. Chem. 2016, 88 (13), 6812–6819. 10.1021/acs.analchem.6b01284. 27239862 PMC4935574

[ref34] Standke S. J.; Colby D. H.; Bensen R. C.; Burgett A. W. G.; Yang Z. Mass Spectrometry Measurement of Single Suspended Cells Using a Combined Cell Manipulation System and a Single-Probe Device. Anal. Chem. 2019, 91 (3), 1738–1742. 10.1021/acs.analchem.8b05774. 30644722 PMC6640145

[ref35] Chen X.; Peng Z.; Yang Z. Metabolomics studies of cell-cell interactions using single cell mass spectrometry combined with fluorescence microscopy. Chem. Sci. 2022, 13 (22), 6687–6695. 10.1039/D2SC02298B. 35756524 PMC9172575

[ref36] Lan Y.; Chen X.; Yang Z. Quantification of Nitric Oxide in Single Cells Using the Single-Probe Mass Spectrometry Technique. Anal. Chem. 2023, 95 (51), 18871–18879. 10.1021/acs.analchem.3c04393. 38092461

[ref37] Liu R.; Zhang G.; Sun M.; Pan X.; Yang Z. Integrating a generalized data analysis workflow with the Single-probe mass spectrometry experiment for single cell metabolomics. Anal. Chim. Acta 2019, 1064, 71–79. 10.1016/j.aca.2019.03.006. 30982520 PMC6579046

[ref38] Nguyen T. D.; Lan Y.; Kane S. S.; Haffner J. J.; Liu R.; McCall L. I.; Yang Z. Single-Cell Mass Spectrometry Enables Insight into Heterogeneity in Infectious Disease. Anal. Chem. 2022, 94 (30), 10567–10572. 10.1021/acs.analchem.2c02279. 35863111 PMC10064790

[ref39] Pan N.; Standke S. J.; Kothapalli N. R.; Sun M.; Bensen R. C.; Burgett A. W. G.; Yang Z. Quantification of Drug Molecules in Live Single Cells Using the Single-Probe Mass Spectrometry Technique. Anal. Chem. 2019, 91 (14), 9018–9024. 10.1021/acs.analchem.9b01311. 31246408 PMC6677389

[ref40] Bensen R. C.; Standke S. J.; Colby D. H.; Kothapalli N. R.; Le-McClain A. T.; Patten M. A.; Tripathi A.; Heinlen J. E.; Yang Z. B.; Burgett A. W. G. Single Cell Mass Spectrometry Quantification of Anticancer Drugs: Proof of Concept in Cancer Patients. Acs Pharmacol Transl 2021, 4 (1), 96–100. 10.1021/acsptsci.0c00156. PMC7887743 33615163

[ref41] Du L.; Risinger A. L.; Mitchell C. A.; You J.; Stamps B. W.; Pan N.; King J. B.; Bopassa J. C.; Judge S. I. V.; Yang Z.; et al. Unique amalgamation of primary and secondary structural elements transform peptaibols into potent bioactive cell-penetrating peptides. Proc. Natl. Acad. Sci. U. S. A. 2017, 114 (43), E8957–E8966. 10.1073/pnas.1707565114. 29073092 PMC5664515

[ref42] Roberts B. L.; Severance Z. C.; Bensen R. C.; Le A. T.; Kothapalli N. R.; Nuñez J. I.; Ma H.; Wu S.; Standke S. J.; Yang Z.; et al. Transient Compound Treatment Induces a Multigenerational Reduction of Oxysterol-Binding Protein (OSBP) Levels and Prophylactic Antiviral Activity. ACS Chem. Biol. 2019, 14 (2), 276–287. 10.1021/acschembio.8b00984. 30576108 PMC6379863

[ref43] Sun M.; Chen X.; Yang Z. Single cell mass spectrometry studies reveal metabolomic features and potential mechanisms of drug-resistant cancer cell lines. Anal. Chim. Acta 2022, 1206, 339761 10.1016/j.aca.2022.339761. 35473873 PMC9046687

[ref44] Liu R. M.; Sun M.; Zhang G. W.; Lan Y. P.; Yang Z. B. Towards early monitoring of chemotherapy-induced drug resistance based on single cell metabolomics: Combining single-probe mass spectrometry with machine learning. Anal. Chim. Acta 2019, 1092, 42–48. 10.1016/j.aca.2019.09.065. 31708031 PMC6878984

[ref45] Liu R.; Zhang G.; Yang Z. Towards rapid prediction of drug-resistant cancer cell phenotypes: single cell mass spectrometry combined with machine learning. Chem. Commun. (Camb) 2019, 55 (5), 616–619. 10.1039/C8CC08296K. 30525135 PMC6640148

[ref46] Chen X.; Sun M.; Yang Z. Single cell mass spectrometry analysis of drug-resistant cancer cells: Metabolomics studies of synergetic effect of combinational treatment - PubMed. Anal. Chim. Acta **04/08/2022**, 2022 1201. DOI: 339621 10.1016/j.aca.2022.339621. 35300794 PMC8933618

[ref47] Liu M.; Zhang Y.; Yang J.; Cui X.; Zhou Z.; Zhan H.; Ding K.; Tian X.; Yang Z.; Fung K.-M. A.; et al. ZIP4 Increases Expression of Transcription Factor ZEB1 to Promote Integrin α3β1 Signaling and Inhibit Expression of the Gemcitabine Transporter ENT1 in Pancreatic Cancer Cells. Gastroenterology 2020, 158 (3), 679–692.e671. 10.1053/j.gastro.2019.10.038. 31711924 PMC7837454

[ref48] Wawrik B.; Bronk D. A.; Baer S. E.; Chi L.; Sun M.; Cooper J. T.; Yang Z. B. Bacterial utilization of creatine in seawater. Aquatic Microbial Ecology 2017, 80 (2), 153–165. 10.3354/ame01850.

[ref49] Sun M.; Yang Z.; Wawrik B. Metabolomic Fingerprints of Individual Algal Cells Using the Single-Probe Mass Spectrometry Technique. Front Plant Sci. 2018, 9, 571 10.3389/fpls.2018.00571. 29760716 PMC5936784

[ref50] Chen X.; Yang Z. Chapter 3 - Biosensors for single-cell metabolomic characterization. In Biosensors for Single-Cell Analysis, Chen J., Lu Y., Eds.; Academic Press, 2022; pp 37–70.

[ref51] Liu R.; Yang Z. Single cell metabolomics using mass spectrometry: Techniques and data analysis. Anal. Chim. Acta 2021, 1143, 124–134. 10.1016/j.aca.2020.11.020. 33384110 PMC7775990

[ref52] Zhang L.; Vertes A. Single-Cell Mass Spectrometry Approaches to Explore Cellular Heterogeneity. Angew. Chem., Int. Ed. Engl. 2018, 57 (17), 4466–4477. 10.1002/anie.201709719. 29218763

[ref53] Herzog R. F. K.; Viehböck F. P. Ion Source for Mass Spectrography. Phys. Rev. 1949, 76 (6), 855–856. 10.1103/PhysRev.76.855.

[ref54] Lanni E. J.; Rubakhin S. S.; Sweedler J. V. Mass spectrometry imaging and profiling of single cells. J. Proteomics 2012, 75 (16), 5036–5051. 10.1016/j.jprot.2012.03.017. 22498881 PMC3419297

[ref55] Liebl H. Ion Microprobe Mass Analyzer. J. Appl. Phys. 1967, 38 (13), 5277–5283. 10.1063/1.1709314.

[ref56] Massonnet P.; Heeren R. M. A. A concise tutorial review of TOF-SIMS based molecular and cellular imaging. Journal of Analytical Atomic Spectrometry 2019, 34 (11), 2217–2228. 10.1039/C9JA00164F.

[ref57] Robinson M. A.; Graham D. J.; Castner D. G. ToF-SIMS depth profiling of cells: z-correction, 3D imaging, and sputter rate of individual NIH/3T3 fibroblasts. Anal. Chem. 2012, 84 (11), 4880–4885. 10.1021/ac300480g. 22530745 PMC3389299

[ref58] Vanbellingen Q. P.; Castellanos A.; Rodriguez-Silva M.; Paudel I.; Chambers J. W.; Fernandez-Lima F. A. Analysis of Chemotherapeutic Drug Delivery at the Single Cell Level Using 3D-MSI-TOF-SIMS. J. Am. Soc. Mass Spectrom. 2016, 27 (12), 2033–2040. 10.1007/s13361-016-1485-y. 27582118 PMC5088064

[ref59] Nunez J.; Renslow R.; Cliff J. B. 3rd; Anderton C. R. NanoSIMS for biological applications: Current practices and analyses. Biointerphases 2018, 13 (3), 03B301 10.1116/1.4993628. 28954518

[ref60] Behrens S.; Kappler A.; Obst M. Linking environmental processes to the in situ functioning of microorganisms by high-resolution secondary ion mass spectrometry (NanoSIMS) and scanning transmission X-ray microscopy (STXM). Environ. Microbiol 2012, 14 (11), 2851–2869. 10.1111/j.1462-2920.2012.02724.x. 22409443

[ref61] Tian H.; Sparvero L. J.; Blenkinsopp P.; Amoscato A. A.; Watkins S. C.; Bayir H.; Kagan V. E.; Winograd N. Secondary-Ion Mass Spectrometry Images Cardiolipins and Phosphatidylethanolamines at the Subcellular Level. Angew. Chem., Int. Ed. Engl. 2019, 58 (10), 3156–3161. 10.1002/anie.201814256. 30680861 PMC6622167

[ref62] Angerer T. B.; Blenkinsopp P.; Fletcher J. S. High energy gas cluster ions for organic and biological analysis by time-of-flight secondary ion mass spectrometry. Int. J. Mass Spectrom. 2015, 377, 591–598. 10.1016/j.ijms.2014.05.015.

[ref63] Zhang W.; Xia X.; Zhang Y.; Peng T.; Yang Q. A novel sample preparation method for ultra-high vacuum (UHV) secondary ion mass spectrometry (SIMS) analysis. Journal of Analytical Atomic Spectrometry 2018, 33 (9), 1559–1563. 10.1039/C8JA00087E.

[ref64] Honig R. E.; Woolston J. R. Laser-Induced Emission of Electrons, Ions, and Neutral Atoms from Solid Surfaces. Appl. Phys. Lett. 1963, 2 (7), 138–139. 10.1063/1.1753812.

[ref65] Tanaka K.; H W.; Ido Y.; Akita S.; Yoshida Y.; Yoshida T.; Matsuo T. Protein and polymer analyses up to m/z 100 000 by laser ionization time-of-flight mass spectrometry. Rapid Commun. Mass Spectrom. 1988, 2 (8), 151–153. 10.1002/rcm.1290020802.

[ref66] Karas M.; Hillenkamp F. Laser desorption ionization of proteins with molecular masses exceeding 10,000 Da. Anal. Chem. 1988, 60 (20), 2299 10.1021/ac00171a028. 3239801

[ref67] Peterson D. S. Matrix-free methods for laser desorption/ionization mass spectrometry. Mass Spectrom Rev. 2007, 26 (1), 19–34. 10.1002/mas.20104. 16967450

[ref68] Keller C.; Maeda J.; Jayaraman D.; Chakraborty S.; Sussman M. R.; Harris J. M.; Ane J. M.; Li L. Comparison of Vacuum MALDI and AP-MALDI Platforms for the Mass Spectrometry Imaging of Metabolites Involved in Salt Stress in Medicago truncatula. Front Plant Sci. 2018, 9, 1238 10.3389/fpls.2018.01238. 30210517 PMC6121006

[ref69] Mandal A.; Singha M.; Addy P. S.; Basak A. Laser desorption ionization mass spectrometry: Recent progress in matrix-free and label-assisted techniques. Mass Spectrom Rev. 2019, 38 (1), 3–21. 10.1002/mas.21545. 29029360

[ref70] Piret G.; Drobecq H.; Coffinier Y.; Melnyk O.; Boukherroub R. Matrix-free laser desorption/ionization mass spectrometry on silicon nanowire arrays prepared by chemical etching of crystalline silicon. Langmuir 2010, 26 (2), 1354–1361. 10.1021/la902266x. 20067318

[ref71] Seino T.; Sato H.; Yamamoto A.; Nemoto A.; Torimura M.; Tao H. Matrix-free laser desorption/ionization-mass spectrometry using self-assembled germanium nanodots. Anal. Chem. 2007, 79 (13), 4827 10.1021/ac062216a. 17542554

[ref72] Addy P. S.; Bhattacharya A.; Mandal S. M.; Basak A. Label-assisted laser desorption/ionization mass spectrometry (LA-LDI-MS): an emerging technique for rapid detection of ubiquitous cis-1,2-diol functionality. RSC Adv. 2014, 4 (87), 46555–46560. 10.1039/C4RA07499H.

[ref73] Mandal A.; Das A. K.; Basak A. Label-assisted laser desorption/ionization mass spectrometry (LA-LDI-MS): use of pyrene aldehyde for detection of biogenic amines, amino acids and peptides. RSC Adv. 2015, 5 (129), 106912–106917. 10.1039/C5RA20678B.

[ref74] Le Pogam P.; Schinkovitz A.; Legouin B.; Le Lamer A. C.; Boustie J.; Richomme P. Matrix-Free UV-Laser Desorption Ionization Mass Spectrometry as a Versatile Approach for Accelerating Dereplication Studies on Lichens. Anal. Chem. 2015, 87 (20), 10421–10428. 10.1021/acs.analchem.5b02531. 26378462

[ref75] Ali A.; Abouleila Y.; Shimizu Y.; Hiyama E.; Emara S.; Mashaghi A.; Hankemeier T. Single-cell metabolomics by mass spectrometry: Advances, challenges, and future applications. TrAC Trends in Analytical Chemistry 2019, 120. DOI: 115436 10.1016/j.trac.2019.02.033.

[ref76] Yang Y.; Huang Y.; Wu J.; Liu N.; Deng J.; Luan T. Single-cell analysis by ambient mass spectrometry. TrAC Trends in Analytical Chemistry 2017, 90, 14–26. 10.1016/j.trac.2017.02.009.

[ref77] Masujima T. Visualized single cell dynamics and analysis of molecular tricks. Anal. Chim. Acta 1999, 400 (1–3), 33 10.1016/S0003-2670(99)00704-7.

[ref78] Mizuno H.; Tsuyama N.; Harada T.; Masujima T. Live single-cell video-mass spectrometry for cellular and subcellular molecular detection and cell classification. J. Mass Spectrom 2008, 43 (12), 1692–1700. 10.1002/jms.1460. 18615771

[ref79] Lorenzo Tejedor M.; Mizuno M.; Tsuyama N.; Harada T.; Masujima T. In situ molecular analysis of plant tissues by live single-cell mass spectrometry. Anal. Chem. 2012, 84, 5221 10.1021/ac202447t. 22243623

[ref80] Fujii T.; Matsuda S.; Tejedor M. L.; Esaki T.; Sakane I.; Mizuno H.; Tsuyama N.; Masujima T. Direct metabolomics for plant cells by live single-cell mass spectrometry. Nat. Protoc. 2015, 10, 1445 10.1038/nprot.2015.084. 26313480

[ref81] Shimizu T.; Miyakawa S.; Esaki T.; Mizuno H.; Masujima T.; Koshiba T.; Seo M. Live single-cell plant hormone analysis by video-mass spectrometry. Plant Cell Physiol. 2015, 56, 1287 10.1093/pcp/pcv042. 25759328

[ref82] Zhang L.; Foreman D. P.; Grant P. A.; Shrestha B.; Moody S. A.; Villiers F.; Kwak J. M.; Vertes A. In situ metabolic analysis of single plant cells by capillary microsampling and electrospray ionization mass spectrometry with ion mobility separation. Analyst 2014, 139 (20), 5079–5085. 10.1039/C4AN01018C. 25109271

[ref83] Zhang L.; Vertes A. Energy Charge, Redox State, and Metabolite Turnover in Single Human Hepatocytes Revealed by Capillary Microsampling Mass Spectrometry. Anal. Chem. 2015, 87 (20), 10397–10405. 10.1021/acs.analchem.5b02502. 26398405

[ref84] Zhang L.; Khattar N.; Kemenes I.; Kemenes G.; Zrinyi Z.; Pirger Z.; Vertes A. Subcellular Peptide Localization in Single Identified Neurons by Capillary Microsampling Mass Spectrometry. Sci. Rep 2018, 8 (1), 12227 10.1038/s41598-018-29704-z. 30111831 PMC6093924

[ref85] Gholipour Y.; Erra-Balsells R.; Hiraoka K.; Nonami H. Living cell manipulation, manageable sampling, and shotgun picoliter electrospray mass spectrometry for profiling metabolites. Anal. Biochem. 2013, 433 (1), 70–78. 10.1016/j.ab.2012.10.001. 23068039

[ref86] Nakashima T.; Wada H.; Morita S.; Erra-Balsells R.; Hiraoka K.; Nonami H. Single-Cell Metabolite Profiling of Stalk and Glandular Cells of Intact Trichomes with Internal Electrode Capillary Pressure Probe Electrospray Ionization Mass Spectrometry. Anal. Chem. 2016, 88 (6), 3049–3057. 10.1021/acs.analchem.5b03366. 26845634

[ref87] Yin R.; Prabhakaran V.; Laskin J. Quantitative Extraction and Mass Spectrometry Analysis at a Single-Cell Level. Anal. Chem. 2018, 90 (13), 7937–7945. 10.1021/acs.analchem.8b00551. 29874047

[ref88] Liu Y.; Shang Y.; Ma Q. Microextraction for ambient ionization mass spectrometry analysis. Advances in Sample Preparation 2022, 3. DOI: 100029 10.1016/j.sampre.2022.100029.

[ref89] Hiraoka K.; Nishidate K.; Mori K.; Asakawa D.; Suzuki S. Development of probe electrospray using a solid needle. Rapid Commun. Mass Spectrom. 2007, 21 (18), 3139 10.1002/rcm.3201. 17708527

[ref90] Gong X.; Zhao Y.; Cai S.; Fu S.; Yang C.; Zhang S.; Zhang X. Single cell analysis with probe ESI-mass spectrometry: detection of metabolites at cellular and subcellular levels. Anal. Chem. 2014, 86 (8), 3809–3816. 10.1021/ac500882e. 24641101

[ref91] Yu Z.; Chen L. C.; Ninomiya S.; Mandal M. K.; Hiraoka K.; Nonami H. Piezoelectric inkjet assisted rapid electrospray ionization mass spectrometric analysis of metabolites in plant single cells via a direct sampling probe. Analyst 2014, 139 (22), 5734–5739. 10.1039/C4AN01068J. 25262850

[ref92] Phelps M.; Hamilton J., Verbeck G. F. Nanomanipulation-coupled nanospray mass spectrometry as an approach for single cell analysis. Rev. Sci. Instrum. 2014, 85. 10.1063/1.4902322 25554307

[ref93] Phelps M. S.; Verbeck G. F. A lipidomics demonstration of the importance of single cell analysis. Anal. Methods 2015, 7, 3668 10.1039/C5AY00379B.

[ref94] Deng J.; Yang Y.; Xu M.; Wang X.; Lin L.; Yao Z. P.; Luan T. Surface-coated probe nanoelectrospray ionization mass spectrometry for analysis of target compounds in individual small organisms. Anal. Chem. 2015, 87 (19), 9923–9930. 10.1021/acs.analchem.5b03110. 26360344

[ref95] Deng J.; Li W.; Yang Q.; Liu Y.; Fang L.; Guo Y.; Guo P.; Lin L.; Yang Y.; Luan T. Biocompatible Surface-Coated Probe for in Vivo, in Situ, and Microscale Lipidomics of Small Biological Organisms and Cells Using Mass Spectrometry. Anal. Chem. 2018, 90 (11), 6936–6944. 10.1021/acs.analchem.8b01218. 29707954

[ref96] Laskin J.; Heath B. S.; Roach P. J.; Cazares L.; Semmes O. J. Tissue imaging using nanospray desorption electrospray ionization mass spectrometry. Anal. Chem. 2012, 84 (1), 141–148. 10.1021/ac2021322. 22098105 PMC3259225

[ref97] Bergman H. M.; Lanekoff I. Profiling and quantifying endogenous molecules in single cells using nano-DESI MS. Analyst 2017, 142 (19), 3639–3647. 10.1039/C7AN00885F. 28835951

[ref98] Ferreira C. R.; Eberlin L. S.; Hallett J. E.; Cooks R. G. Single oocyte and single embryo lipid analysis by desorption electrospray ionization mass spectrometry. J. Mass Spectrom 2012, 47 (1), 29–33. 10.1002/jms.2022. 22282086

[ref99] Colwell N.; Chen D.; Yang Z. Achieving Single-cell Resolution via Desorption Electrospray Ionization Mass Spectrometry Imaging (DESI-MSI). ChemRxiv 2024, 10.26434/chemrxiv-2024-826pr. PMC13084628 41911401

[ref100] Shrestha B.; Vertes A. In Situ Metabolic Profiling of Single Cells by Laser Ablation Electrospray Ionization Mass Spectrometry. Anal. Chem. 2009, 81 (20), 8265 10.1021/ac901525g. 19824712

[ref101] Nemes P.; Vertes A. Laser Ablation Electrospray Ionization for Atmospheric Pressure, in Vivo, and Imaging Mass Spectrometry. Anal. Chem. 2007, 79 (21), 8098 10.1021/ac071181r. 17900146

[ref102] Lee J. K.; Jansson E. T.; Nam H. G.; Zare R. N. High-Resolution Live-Cell Imaging and Analysis by Laser Desorption/Ionization Droplet Delivery Mass Spectrometry. Anal. Chem. 2016, 88 (10), 5453–5461. 10.1021/acs.analchem.6b00881. 27110027 PMC5446058

[ref103] Schober Y.; Guenther S.; Spengler B.; Rompp A. Single cell matrix-assisted laser desorption/ionization mass spectrometry imaging. Anal. Chem. 2012, 84 (15), 6293–6297. 10.1021/ac301337h. 22816738

[ref104] Pan N.; Rao W.; Yang Z. Single-Probe Mass Spectrometry Analysis of Metabolites in Single Cells - PubMed. Methods Mol. Biol. 2020, 2064. DOI: 61 71 10.1007/978-1-4939-9831-9_5. 31565766

[ref105] Standke S. J.; Colby D. H.; Bensen R. C.; Burgett A. W. G.; Yang Z. Integrated Cell Manipulation Platform Coupled with the Single-probe for Mass Spectrometry Analysis of Drugs and Metabolites in Single Suspension Cells. J. Vis Exp 2019, ( (148), ). DOI: 10.3791/59875. PMC6677261 31282898

[ref106] Ho C. S.; Lam C. W.; Chan M. H.; Cheung R. C.; Law L. K.; Lit L. C.; Ng K. F.; Suen M. W.; Tai H. L. Electrospray ionisation mass spectrometry: principles and clinical applications. Clin. Biochem. Rev. 2003, 24 (1), 3–12. 18568044 PMC1853331

[ref107] Wije Munige S.; Bhusal D.; Peng Z.; Chen D.; Yang Z. Developing Cell Quenching Method to Facilitate Single Cell Mass Spectrometry Metabolomics Studies 2024, 10.26434/chemrxiv-2024-mmf9z. PMC12117403 40443889

[ref108] Zou Z.; Peng Z.; Bhusal D.; Wije Munige S.; Yang Z. MassLite: An integrated python platform for single cell mass spectrometry metabolomics data pretreatment with graphical user interface and advanced peak alignment method. Anal. Chim. Acta 2024, 1325, 343124 10.1016/j.aca.2024.343124. 39244309 PMC11462640

[ref109] Yao S.; Nguyen T. D.; Lan Y.; Yang W.; Chen D.; Shao Y.; Yang Z. MetaPhenotype: A Transferable Meta-Learning Model for Single-Cell Mass Spectrometry-Based Cell Phenotype Prediction Using Limited Number of Cells. Anal. Chem. 2024, 96, 19238 10.1021/acs.analchem.4c02038. 39570119 PMC11673283

[ref110] Rao W.; Pan N.; Yang Z. High Resolution Tissue Imaging Using the Single-probe Mass Spectrometry under Ambient Conditions. J. Am. Soc. Mass Spectrom. 2015, 26 (6), 986–993. 10.1007/s13361-015-1091-4. 25804891

[ref111] Wheeler K.; Gosmanov C.; Jimenez Sandoval M.; Yang Z.; McCall L.-I. Frontiers in mass spectrometry-based spatial metabolomics: Current applications and challenges in the context of biomedical research. TrAC–Trends Anal. Chem. 2024, 175, 117713, 10.1016/j.trac.2024.117713. PMC11905388 40094101

[ref112] Tian X.; Zou Z.; Yang Z. Extract Metabolomic Information from Mass Spectrometry Images Using Advanced Data Analysis. In Mass Spectrometry Imaging of Small Molecules: Methods and Protocols, Lee Y.-J., Ed.; Springer US, 2022; pp 253–272. 10.1007/978-1-0716-2030-4_18 34902154

[ref113] Luo S.; Wu Q.; Li Y.; Lu H. Per-pixel absolute quantitation for mass spectrometry imaging of endogenous lipidomes by model prediction of mass transfer kinetics in single-probe-based ambient liquid extraction. Talanta 2021, 234, 122654 10.1016/j.talanta.2021.122654. 34364463

[ref114] Luo S.; Zhao Z.; Wu Q.; Wang Y.; Lu H. Porous Graphitic Carbon-Based Imprint Mass Spectrometry Imaging with an Ambient Liquid Extraction Technique for Enhancing Coverage of Glycerolipids and Sphingolipids in Brain Tissue. Anal. Chem. 2022, 94 (40), 13753–13761. 10.1021/acs.analchem.2c01991. 36173256

[ref115] Lei J.; Zhao Z.; Wu Q.; Lu H. Graphene Oxide/TiO(2) Nanocomposite-Assisted Two-Step Ambient Liquid Extraction Mass Spectrometry Imaging for Comprehensively Enhancing Lipid Coverage in Spatial Lipidomics. Anal. Chem. 2024, 96, 19456 10.1021/acs.analchem.4c03955. 39593233

[ref116] Lv Y.; Zhao Z.; Long Z.; Yu C.; Lu H.; Wu Q. Lewis Acidic Metal-Organic Framework Assisted Ambient Liquid Extraction Mass Spectrometry Imaging for Enhancing the Coverage of Poorly Ionizable Lipids in Brain Tissue. Anal. Chem. 2024, 96 (3), 1073–1083. 10.1021/acs.analchem.3c03690. 38206976

[ref117] Zhou Y.; Zhao Z.; Wu Q.; Lei J.; Cui H.; Pan J.; Li R.; Lu H. Photoinduced Online Enrichment-Deglycosylation of Glycolipids for Enhancing Lipid Coverage and Identification in Single-Cell Mass Spectrometry. Anal. Chem. 2024, 96 (44), 17576–17585. 10.1021/acs.analchem.4c03343. 39435868

[ref118] Zhang L.; Xu T.; Zhang J.; Wong S. C. C.; Ritchie M.; Hou H. W.; Wang Y. Single Cell Metabolite Detection Using Inertial Microfluidics-Assisted Ion Mobility Mass Spectrometry. Anal. Chem. 2021, 93 (30), 10462–10468. 10.1021/acs.analchem.1c00106. 34289696

[ref119] Zhang D.; Qiao L. Microfluidics Coupled Mass Spectrometry for Single Cell Multi-Omics. Small Methods 2024, 8 ( (1), ). DOI: 10.1002/smtd.202301179. 37840412

[ref120] Feng D.; Xu T.; Li H.; Shi X.; Xu G. Single-cell Metabolomics Analysis by Microfluidics and Mass Spectrometry: Recent New Advances. J. Anal. Testing 2020, 4 (3), 198–209. 10.1007/s41664-020-00138-9.

[ref121] Mellors J. S.; Jorabchi K.; Smith L. M.; Ramsey J. M. Integrated Microfluidic Device for Automated Single Cell Analysis Using Electrophoretic Separation and Electrospray Ionization Mass Spectrometry. Anal. Chem. 2010, 82 (3), 967 10.1021/ac902218y. 20058879 PMC2836921

[ref122] Yin H.; Marshall D. Microfluidics for single cell analysis. Curr. Opin. Biotechnol. 2012, 23 (1), 110–119. 10.1016/j.copbio.2011.11.002. 22133547

